# Spirit of the Foreign Periodicals

**Published:** 1834-07-01

**Authors:** 


### Spirit of the Foreign Periodicals, &c.

### Vaccination in Italy.

Dr. Festler has published in the January Number of the Annali Universali an instructive report on the variolous epidemic which prevailed last year in the district of Albignasego, of which he was medical superintendant. It is most gratifying to observe, that the physicians of all the countries which have hitherto adopted vaccination, are unanimous in the judgment which they have formed of the striking advantages of this great boon: admitting, indeed, that it is not invariably a secure defence against the inroads of small-pox, they are forced by the love of truth to proclaim, that few, very few of those who have been properly vaccinated are susceptible of the infection of the variolous poison; and that moreover (a most important fact) in such as have caught the disease after vaccination, it is almost always mild and easily tractable.

The testimony of Dr. Festler cannot possibly be gainsayed; not one of his vaccinated patients died, and with the exception of a single case, the symptoms were those rather of a varicellous or of a varioloid affection, than of genuine small-pox. Not a few who caught the disease in the natural way fell victims to it. It is a circumstance worthy of mention, that the first case of all, at least as far as Dr. F. could ascertain, occurred in a female who had been vaccinated when young: in her it was varioloid, but as several living in the same house had never been subjected to the cow-pox, they were seized with the actual variola, and as we have said, many died. It appears unnecessary to trace the details of individual cases; the general results only it is of consequence to make known; and first with regard to the different forms which the disease put on, when it attacked those who had been previously vaccinated. They may be reduced to three; viz. varicella, varioloid, and lastly the genuine variola. We shall say a few words on each.

1. *Varicella.* The smallest number of cases, and these too were of the mildest form, occurred in the youngest patients, or in those who most recently had been vaccinated. After a slight pyrexia of one, two, or three days' standing, a few red spots, interspersed with small tubercles of the natural colour of the skin, made their appearance on different parts of the body; the feverish symptoms thereon ceased; the spots became vesicles or pustules, and these dried away almost as soon as formed; the tubercles on the other hand seemed like abortive variolous papulæ, and continued for a few days longer, and then gradually scaled off; the exfoliation affecting only the cuticle. In 8, or 10 days at most, from the invasion of the disease, the patients were quite well.

2. *The Varioloid,* — affected chiefly those above 15 years of age or thereabouts; not but that many at and above this period of life exhibited nothing but the mild varicella; all that we intend to say, is, that very few cases of varioloid occurred in those who were younger. As we have already stated, the younger the patients, the less susceptible were they of the infection, and the milder generally was the disease. The greater number therefore of the cases were of the varioloid type. The eruptive fever was more severe, and was frequently accompanied with some irritation in the throat and trachea, as in coryza and slight bronchitis. On the 3d, 4th, or 5th day, a rash, like that of measles or of scarlatina, appeared; this was soon succeeded by distinct papulæ, or tubercles, which in a day or two became vesicular; the lymph of these vesicles, at first clear and like water, gradually thickened and assumed a yellowish colour; some of them remained pointed, others became flat, or depressed in the centre; the former contained least lymph, and, upon drying, they left behind the colourless tubercles, which we alluded to when speaking of varicella.

The usual period of the desiccation of the pustules was from the 11th to the 14th day, counting from the commencement of the disease. Sometimes, although rarely, a few pustules on the face or chest remained longer, suppurating slowly, but not attended with any febrile re-action. On the 18th day, almost all the patients had quite recovered; no pits were left behind, only some faintly-red spots or blotches. The eruption of the varioloid was never distinctly or generally confluent—the colour of the crusts or scabs was an ashen or dirty-yellow colour; when once thrown off, they were never renewed.

3. *Variola.* Only one case of genuine small-pox, after vaccination, occurred; and, although, severe, the patient ultimately did well. In it the third stage, or that of the maturation of the pustules, was well marked; the pyrexia never ceased entirely, and was followed by a regular secondary fever, which continued until the end of the third week.

These three forms of eruptive disease we consider to be produced by the same poison, acting on differently-disposed constitutions; each is capable of propagating either of the three forms, so that, from a mild varicella, a person may be infected with a severe variola, and, on the other hand, the worst form of small-pox may communicate only a slight cow-pox; all depends on the susceptibility of the individual exposed, and the susceptibility appears to be proportionate to the interval between the vaccination and the exposure to the infection.

Is not, therefore, the line of conduct which ought to be pursued obvious to all? Whenever a case, whether of varicella, varioloid, or variola occurs, the patient, and every inmate of the same dwelling, should be confined to their house, and prevented, under a severe penalty, from associating with others. It is only by such a rigorous and wholesome enactment that we can hope to crush this many-headed monster. At the same time, vaccination ought to be authoritatively, unconditionally, and exclusively promoted by all governments.

On the subject of *re-vaccination*, Dr. Festler states that he has himself re-vaccinated upwards of 50 persons, of different ages, from 2 to 32 years. Of these, not above 20 were affected specifically with the virus. Indeed, the phenomena varied so considerably in different individuals, that we may conveniently arrange them in three classes, corresponding somewhat with the threefold division of varicella, varioloid, and variola.—1, Some resisted the operation of the poison altogether, and the punctures on the arm were not more inflamed than if they had been made with a clean lancet; two-thirds of the whole number, as we have just now stated, belonged to this class. 2, In others, the punctures were more inflamed, and on the fourth day or so, a pointed tubercle or small boil appeared on each—the suppuration was quite superficial, and, on the fifth or sixth day, had dried to a rough scab or crust, accompanied with much troublesome itching; in two or three more, the crust fell off without leaving any scar. 3, And, in a third class, the development and progress of the tubercle resembled more nearly, but by no means regularly or perfectly, the maturation and decay of the genuine vaccine pustule. The suppuration did not affect the subjacent cellular texture—the red areola was more circumscribed, and of a paler colour; the pustule was visibly umbilicated, the crust did not exhibit the concentric striæ and circles, it fell off sooner, and the cicatrix was not dotted as after genuine vaccination, but was rather like to the scar of a slight burn or scald. This form may, therefore, be compared with the eruption of the varioloid; the specific action of the virus does not penetrate deeper than the cuticle, whereas, in the true variolous and vaccine pustules, the subcutaneous and cellular texture are equally involved.

In order to satisfy himself that the matter of the imperfect pustule of a revaccinated person has all the specific agency of genuine cow-pox, Dr. Festler inserted some into the arms of infants who had never been vaccinated, and the consequence was, that perfect and complete vaccine pustules were induced, so that it is quite apparent that the modified effects, in the first instance, depended upon the state of the constitution of the individual, and not upon the quality of the virus. Dr. Festler repeated the operation a third time in the case of his own wife, and the pustule which, on the former occasion, belonged to the third division above described, was now more irregular and imperfect, as in the second division. The susceptibility has, therefore, he thinks, become less and less, although it may still exist in a small degree. Neither Madame, nor any other of his revaccinated patients, suffered at all from the variolous epidemic, although frequently exposed to its immediate influence.—*Annali Universali, Jan.* 1833.

---

Thymi in Homine, ac per Seriem Animalium Descriptio Anatomica, Pathologica, et Physiologica, iconibus ad Naturam Delineatis, illustrata. Auct. F. C. Haugsted. Copenhague, 1832.

Little attention has hitherto been paid to the subject of the anatomy, healthy as well as morbid, of the thymus gland. With the exception, and we admit that to be no unimportant one, of the late labours of Sir A. Cooper, medical men seem to have almost forgotten that there was such an organ in the body, or, at least, to have quietly acquiesced in the assurance, that all enquiries as to its structure, functions, and morbid changes must be fruitless and of no avail; so powerful is the influence of mere habit in matters of science, as well as in every occurrence of life. It may be useful, therefore, to attempt to chase away this incubus of indolent ignorance, and we know of no better way to effect our purpose, than by presenting to the medical public an analytical review of M. Haugsted's elaborate treatise, published two years ago at Copenhagen. As a general remark, applicable to almost all the pathological changes to which the thymus is subject, it may be stated that the symptoms which they occasion during the life of the patient indicate some disturbance of the respiratory functions, and that, associated with this disturbance, there is a considerable emaciation of the body, especially in very young children. Mr. Hood, indeed, in the Edinburgh Journal of Medical Science for 1826, has suggested that hydrocephalus may not unfrequently be occasioned by diseased enlargements of the thymus gland, in consequence of the pressure occasioned on the jugular and subclavian veins, and of the impeded return of the blood thereby induced. But this idea is not supported by any data which we can depend upon. In our enumeration of the abnormal conditions of this organ, we shall first arrange those which may be termed "congenital anomalies of formation," or "vitia evolutionis"—such as its entire absence, unusual smallness or largeness, increase in the number of the lobes, &c.; and afterwards the alterations of structure resulting from disease, as inflammation, suppuration, scirrhus, tubercles, tumors, ossification, &c.

1. *Absence of the Thymus.*—No well-authenticated instance of the entire want of this gland, in a child otherwise perfect, is perhaps on record. In anencephalous monsters, its absence is not infrequent. Meckel most distinctly states that, in all the dissections which he has made, he has never once met with an instance of absence of the thymus gland, except in truly anencephalous monsters. Winslow had previously made the same remark, and has alluded to the thymus and the supra-renal gland also, being awanting, in children born without any cerebrum.

In our descriptions, we must carefully distinguish between such monsters as are truly acephalous, or rather anencephalous, and such as are "des acephales faux," or, as they ought rather to be named, "acranial," the anomaly, in these latter, consisting in the absence of the vault of the cranium only, and not of the cerebrum within. Brodie met with a singular case, in which not only the thymus, but also the heart, pleura, and liver were awanting in a seven-month fœtus, not acephalous.

As to the remark of Wrisburg, that he could not find any traces of the gland in a fœtus, which he considered to be not more that ten weeks old, it is not at all surprising; for the very good reason, that it is only during the third month that the gland begins to be formed.

2. *Increase of the Number of the Lobes.*—The multiplication of the lobes of the thymus appears to be not unfrequent in acephalous monsters. Thus Otto, in his description of such a monster, has remarked—"the viscera of the chest did not exhibit any unusual appearances except the thymus gland, which was found to be composed of five long narrow lobules, and to be of a much larger size than is common." Probably, in all such cases, there is no actual augmentation in the number of the lobules; but only the connecting cellular substance is unusually loose, or is altogether deficient: and as the gland, during the early stages of its formation, is always more or less divided into distinct portions, Meckel is inclined to attribute its lobulated condition in the more advanced being to an arrest of development.

3. *Extreme Smallness of the Thymus.*—In acephalous or acranial fœtuses the gland is not commonly wanting altogether, but is frequently much smaller than usual. Otto, Meckel, and M. Reynard have furnished examples of this anomaly. Its occurrence is however by no means constant in these monsters; for occasionally the gland has been found exceeding its ordinary dimensions in them. In an acranial fœtus of eight months examined by Otto, and in which the brain was invested with the common integuments only, the thymus gland was composed of three large, irregular, and divided lobes, which occupied all the upper portion of the anterior mediastinum, stretching considerably on each side, and reaching up as high as the lower edge of the thyroid gland;—its colour was reddish-white. In another acranial fœtus the thymus exhibited five lobes of considerable dimensions.—Kilch, in his contributions to pathological anatomy, published at Berlin in 1813, mentions the case of an anencephalous monster, in which the place of the brain was occupied by 10 or 15 hydatids, invested with the integuments only; and in which the thymus was of an unusual volume, while the glandulæ supra-renales were smaller than is common. From these statements we perceive that it is impossible to establish any accurate inferences respecting the uniformity of an impeded or arrested development of the thymus gland, when the cerebral mass is either imperfectly formed, or has undergone intra-uterine degeneration.

It may however be noticed that, excepting in the cases of acephalous and anencephalous monsters, not one instance is on record of the thymus having been found of extremely or morbidly small dimensions. We have already alluded to the error committed by Wrisberg in his "Descriptio Anatomica Embryonis," of considering the very diminutive size of the gland in a three-months' embryo as an abnormal condition.

4. *Enlargement of the Thymus.*—This pathological state of the gland has been noticed by many anatomists; and the more so, because a certain train of symptoms, indicative of disturbed respiration, very usually accompanies its existence. We shall briefly allude to a few illustrative examples. At the associate meeting of naturalists, held at Heidelberg in 1829, Dr. Kopp read a memoir on one form of asthma occurring in infants, which he traced to an hypertrophy of the thymus gland; the result of this morbid state being a certain degree of compression on the trachea and bronchi. He designated the disease "asthma thymicum," supposing that it had not been noticed by previous observers. But this is quite an error of ignorance. No words can be more explicit than those of Frank, in his Epitome de Curand. Hom. Morb.

" In asthmate, ut nominant, puerili, glandulas bronchiales præter sanitatis modum turgidas, maxime vero thymum insigniter tumefactum invenerunt anatomici." The description which Dr. Kopp has given of the symptoms is very copious and full. The dyspnœa, we are told, usually occurs in paroxysms, and these are more frequent when the infant is sucking, or has just awaken from sleep, than at other times. The explanation of this is easy;—during sleep, the inspirations are less profound, and the lungs being therefore less expanded, more room is left for the enlarged thymus. Whenever the child awakes, it suddenly draws in a deep breath; the thymus resists the full dilatation of the lungs; and a paroxysm of dyspnœa is thus induced. A fit of crying has nearly the same effect; and in short, any effort which is accompanied by a sudden and rapid inspiration.

Dr. Kopp alludes to the increase of size, which the thymus very generally acquires in hybernating animals during their state of inaction, and attributes it to the circumstance of the lungs being but very imperfectly dilated, and thereby occupying less than their usual space within the thorax; but the explanation is more ingenious than satisfactory; for it appears that in this tribe of animals the organs which acquire an augmentation of volume during their hybernating sleep, are situated rather out of, than within the thoracic cavity.

The mere circumstance of the thymus being found enlarged in those cases alluded to by Dr. K. is not sufficient in itself to justify the conclusion, that all the asthmatic symptoms are induced by the compression thereby exerted on the respiratory organs.

It is not common to find an hypertrophied state of the gland alone, and unassociated with some lesions of other organs, especially of the heart and lungs. Such being the case, we can readily understand the cause of the respiratory distress; and it may reasonably be disputed, whether the enlargement of the gland is the cause or the result of the other lesions. Caspari and Pagenstecher have been led by their investigations to regard the increased volume of the thymus, in those cases included under Kopp's " asthma thymicum," as induced by, or as the consequence of the dyspnœa, the true cause of which is, in their opinion, an affection of the par vagum. We cannot, however, reasonably doubt that the thymus, when it has acquired a considerable enlargement, must of a necesssity offer some degree of impediment to the free dilatation of the lungs, just in the same way as the liver, spleen, &c., when their dimensions are preternaturally increased, give rise to symptoms of distressed breathing. One difficulty, not easily explained, will readily suggest itself to the reader. How is it that the dyspnœa comes on in paroxysms when the exciting cause is not an occasional but a permanent one? Surely there is no reason for believing that the symptoms are referable to a periodic congestion of humours in the gland. The following case is important, as it shews that an abnormal enlargement of the thymus is not always accompanied with dyspnœa.

A girl, seven years of age, was admitted into the hospital at Copenhagen in October 1830. For the preceding six months she had been afflicted with severe headaches, to which were now added sickness and occasional vomitings. The looks of the child were heavy and dull, and indicated, in connexion with the other symptoms, the existence of hydrocephalic disease. The breathing had been all along, and still continued to be, quite easy and unobstructed. Calomel and digitalis had been exhibited freely; the tartar-emetic ointment applied to the scalp, and a blister to the nape of the neck. No relief was obtained from any of these remedies, and the young patient died on the sixth day after her admission. On dissection, there was found a considerable effusion into the cerebral ventricles. The thymus gland was of unusual dimensions, and was much larger than in any one case of asthma thymicum related by Dr. Kopp himself. It weighed five ounces. The right lobe, which was the one most enlarged, extended in front of the right lung, to which it adhered as far as the angle of the ribs;—it measured four inches in length, two in breadth, and was nearly an inch and a half in thickness. Its texture had become degenerated into a granular or tuberculous mass, which presented no traces of any cells or of lobules. The upper lobes of the lungs also exhibited tuberculous changes.—The thyroid gland and the capsulæ supra-renales were normal.

The anatomist Riolan entertained the whimsical idea that the paroxysms of hysteric suffocation were induced by a temporary intumescence of the thymus compressing the respiratory organs; and he explained the more frequent occurrence of such attacks in females than in those of the other sex, by the larger proportional size of the gland in the former. It is surely quite unnecessary to expose the absurdity of this doctrine. According to the researches of some pathologists, an abnormal development of the thymus has in numerous instances induced sudden and fatal suffocation in very young infants; and Schallgrubber is of opinion, that mothers and nurses have been frequently accused of having either wilfully or through negligence caused the death of the child, when the real cause of the accident was what we have just mentioned. If his opinion be confirmed, it must be of great importance in legal medicine to attend to this occasional source of sudden death. Certainly in most of the cases of enlarged thymus there is, as has been already remarked, a coexistent morbid affection of some of the other thoracic viscera; yet, although it cannot be regarded as the sole agent of mischief, it is quite probable that the compression from the magnified gland may act as a direct or immediate cause of inducing the train of morbid phenomena. In several of the examples recorded by Fleischmann, Sandifort, Morgagni, Ribes, Kopp, and others, there was coexistent morbus cæruleus, and dissection revealed an open state of the foramen ovale of the heart;—in other examples, however, the abnormal size of the thymus was not associated with any affection either of the heart or of the lungs. Thus Meckel has described the case of a child two years old, who died of hydrocephalus, and in whom the only trace of thoracic disease was an enlarged state of the thymus. Rust, in his Magazine for 1826, has detailed the particulars of a case of sudden suffocation in a child;—it had previously enjoyed perfect and uninterrupted health till within a short time before its death. The earliest symptoms were violent cough and dyspnœa, recurring however at intervals of several days at first, and the fits gradually becoming more frequent and severe; so that towards the close they were renewed several times in the course of an hour;—in one of these the child suddenly expired. On dissection the thymus was found of unusual size, measuring three inches and a half in length, and one and a half in breadth. Its texture was firmer and more solid than natural, and it was so situated, that it might have exerted considerable pressure on the arteria innominata. Dr. Eck, of Berlin, has described in the same journal the case of his own child, which died of what he considered to be the "asthma of Dr. Millar." The symptoms were nearly the same as Dr. Kopp has enumerated as characteristic of the "asthma thymicum." After death, the gland was found to be much hypertrophied, and to have pushed the lungs to the posterior part of the thorax. The heart, lungs, and air-passages were healthy; and no effusion existed within the cranium. In our own practice, we (the Rev.) met with a case not long ago, which, as it has reference to the present subject, may deserve to be recorded here.

A fine, plump, well-formed child, about 18 months old, suddenly expired in a fit of convulsions. There had been six or eight similar attacks before, occurring at intervals of one or two months. They were attributed to the irritation of teething, and had generally soon given way to the continued application of a stream of cold water on the head, while the feet and lower part of the body were immersed in a warm-bath. No symptom of disturbed respiration, except a very slight croupy noise occasionally, had at any time been noticed.

On dissection all the abdominal and thoracic viscera were found presenting a most healthy appearance; the thymus gland however was of an unusual size, measuring about four inches in length, and weighing at least 2½ or 3 ounces. No trace of any cerebral disease could be detected.

But it is not in young children only that an hypertrophied state of the thymus has been discovered; it has been met with occasionally in adults and in old people; sometimes along with, at other times without, an affection of the heart or lungs. Unfortunately the descriptions of authors on this topic have been exceedingly vague and unexplicit; the texture of the enlarged gland is seldom specified, and we are left in utter uncertainty whether the organ had merely retained its infantile condition, or whether it had assumed other appearances from a more late deposition of new matter, (perhaps in proportion as the respiratory disturbance increased,) or lastly, whether the suspected enlargement was not in reality a morbid growth merely occupying the site of the thymus.

In the beginning of the last century Cowper and Budœus recorded two cases which occurred in men who were about 30 years of age. The particulars of one of these are the following. The patient had laboured under dyspnœa from his infancy, and at length he died quite suffocated.

The pulmonary parenchyma was found studded with tuberculous deposit; the thymus gland had acquired an immense size, was indurated, scirrhous and cartilaginous, in part resembling the texture of a scrofulous or steatomatous tumor: it adhered very strongly to the large vascular trunks; occupied all the upper portion of the thoracic cavity, and had very obviously presented a great impediment to the free dilatation of the lungs. The heart was enlarged, and the pericardium was found distended with fluid.

Meckel (primus) describes the history of a soldier, 26 years of age, who had long suffered from paroxysms of dreadful anxiety and dyspnœa: at first their recurrence was at considerable intervals, but afterwards they became much more frequent. In the upper part of the cavity of the anterior mediastinum there existed two large lobes of the thymus; each of these was three and a half inches long, and from six to ten lines in breadth; their texture was nearly the same as in the fœtus: they were cellular, and might be distended by blowing air into them; several considerable sized arteries and veins derived from the internal mammaries and inferior thyroids were distributed upon their substance: their colour was a reddish white; very little adipose matter existed in the surrounding cellular substance. Tozetti in his "Raccolta di Opusculi Medico-practici," has recorded a case in which the thymus was found of extraordinary dimensions. The patient, a countryman, 57 years of age, had laboured during 20 years under occasional dyspnœa, the paroxysms had become gradually more frequent, and had been accompanied with dropsy of the chest and abdomen. The thymus measured four inches in length, three in breadth, and two in thickness; and its weight amounted to nine ounces. The texture was more solid and fleshy-looking than is natural. To this enlarged mass there adhered a smaller tumor, which seemed to be of a scrofulous character. Tozetti attributes the dropsical effusions to the compression which the enlarged thymus exerted on the arterial and venous trunk. Hufeland, in his Journal of Practical Medicine, for the year 1806, has related a case of fatal angina pectoris, in which the only morbid lesion detected after death was an enormous fatty-like enlargement of the thymus gland, which occupied the whole of the anterior mediastinum.

5. *Inflammation of the Thymus—Thymitis.* It must be acknowledged that this disease has seldom been ascertained, or even suspected during the life of the patient; but the occasional occurrence of collections of purulent matter being found within its substance is sufficient to prove the existence of an antecedent phlegmasia. Sauvages indeed has enumerated in his nosology the symptoms which he considered as pathognomonic of this state; these are, a certain degree of impediment to deglutition and to speech, vomiting after eating, dyspnœa, and the external appearance of a tumefaction at the lower part of the neck. Portal, in his edition of Lieutaud, mentions the case of a child, two years of age, who died of convulsions. On dissection there was found an effusion into the pleura and pericardium; the thymus was prodigiously increased in volume, and on cutting it through a large cyst full of a blackish sanies was discovered. But the far greater number of examples of suppuration occurring in the substance of the thymus gland have been preceded by the existence of tuberculous matter, which very generally at the same time is found in the texture of the lungs. This tubercular degeneration is most commonly met with in strumous children; the substance of the gland becomes more dense and solid, and all traces of the cells gradually disappear, so that when the disease has existed for a considerable time, the thymus is found to present the appearance of a conglobate gland. The shameful inaccuracy of medical language has sometimes designated this morbid change by the term "scirrhus."

M. Cruveilhier has seen the thymus in a young infant, filled with tubercles in a state of suppuration, such as we observe in the lungs, in the last stage of phthisis.

Portal has alluded to the frequency of tubercles being found in the thymus and anterior mediastinum in rachitic children; and Lieutaud has recorded several instances of the same disease in adults who had died of consumption, or of other pulmonary affections.

In Haller's disputations we find the case of a young student who, after having suffered for a considerable time from attacks of hæmoptysis, was seized with a severe pain at the lower and anterior part of the neck, accompanied at first with cough, and afterwards with difficulty of breathing and purulent expectoration. On dissection the lungs were found loaded with tubercles, and the thymus had acquired an unusual size, and contained within, a cavity full of pus, and which communicated with the trachea.

6. *Ossification and Calculi of the Thymus.* As in the conglobate and conglomerate glands in different parts of the body, we occasionally find calcareous concretions which have been attributed by many to the induration, or solidification of their peculiar juices, so in the case of the thymus, similar changes have been observed. It is very doubtful, however, whether a truly ossific deposit has ever been discovered in the substance of the thymus, although numerous cases of such a change are on record; for the truth is, that medical writers have frequently applied the term "ossification" to every sort of solid and dense concretion, whatever be its nature and characters. There is however this important distinction between a bony and a calculous deposit, that the former contains in addition to its phosphate of lime, an animal gelatine which is never found in any calculus. And, again, all ossific formations are organised substances, being nourished by their proper bloodvessels, and susceptible of absorption; whereas calculous concretions are completely inorganic. Some authors have insisted too much upon the mere differences of consistence in the two deposits; but this is a very uncertain criterion to distinguish them by. Chemical analysis is the only sure test. Vater, Harder, Hoffmann, and others, have recorded examples of calcareous degeneration of the thymus.

---

### Clinical Researches on the Medicinal Effects of Digitalis.

The reports of medical men on the powers and properties of this potent drug have been strangely conflicting and inconsistent with each other. Cullen affirmed that the direct physiological operation of foxglove is to weaken and retard the action of the heart and arteries ; Dr. Sanders has on the other hand maintained that it first quickens the pulse; and lastly, Orfila and others have not obtained any steady or uniform results, either the one way or the other. Equally discordant have been the statements of different authors respecting the therapeutic effects of digitalis. If we are to believe Drs. Bidault and Currie, it is of great efficacy in all inflammatory diseases ;— Clutterbuck considered it as the only specific against fever ;—asthma and epilepsy have been said to be under its immediate control ; Darwin, Fowler, and Beddoes would lead us to suppose that it is able to cure hæmoptysis and even phthisis, and Hufeland has pompously eulogized it as the most heroic of all anti-scrofulous remedies ! ! That it possesses beneficial powers in quickening the action of the absorbents, and in increasing the flow of urine in certain cases of dropsy will be disputed by few ; but that it has cured encysted dropsies of the ovarium, must surely be the mere fantasy of a fond admirer.

The following researches were instituted, and very carefully pursued, for the purpose of arriving if possible at some satisfactory conclusions respecting the physiological and therapeutic effects of digitalis. It was exhibited sometimes in the form of powder recently prepared, and possessing all the aromatic qualities of the fresh herb ; at other times, of a watery or alcoholic extract ; and lastly of infusion. The extracts were obtained in the following manner, according to the directions of Professor Soubeiran, the director of the Central Pharmacy of Hospitals. The juice of the fresh herb, procured by expression, was gently heated, for the purpose of coagulating the vegetable albumen ; it was then filtered, and evaporated in a water bath to a proper consistence. The alcoholic extract was obtained by slowly evaporating a concentrated tincture of the leaves.

It may be mentioned that most of the following cases have been derived from the clinique of Professor Andral, at the Hôpital de la Pitié.

EXPERIMENTS WITH THE POWDER.

CASE 1. *Hypertrophy and Dilatation of the Heart*—48 *grains of Digitalis in* 6 *days.*

*March* 24*th.* A woman, aged 60, was admitted with the following symptoms. Impulsions of the heart very strong ; upon any effort of the breathing as in coughing, blowing the nose, &c. they become so violent, that they caused her to faint away, and the extremities to become cold and benumbed. The pulse is 68 ; the number of respirations in the minute only 14. She suffers much from headache, noises in the ears, confusion of sight, with a pricking sensation in the eyes, pain in the region of the kidneys, and general anasarca of the lower limbs. These symptoms are of three months' duration ; but it is nearly a twelvemonth since she had an attack of severe dyspnœa, from which period she dates the commencement of her illness. She was ordered four grains of digitalis to be taken in the course of the day.

25*th. Pulse* 92—*Respirations* 38—8 *grains of digitalis ordered.*

This quantity was continued every day until the 30th without any very appreciable results on the circulatory and digestive functions. The medicine was then discontinued, and the patient kept upon a light emollient diet. On the 4th of April she was seized with almost constant fits of fainting, and with distressing anxiety. Pulse 96, and the palpitations stronger than ever. Respirations 36. A more decided antiphlogistic regimen was now adopted, but with little relief to the symptoms.

*Remarks.* No very striking effects were produced on any of the functions in this case. The pulsations of the heart were little influenced ; the urine was not affected at all.

CASE 2.—*Hypertrophy of the Heart* —60 *grains taken in* 6 *days.*

A shoemaker, 59 years of age, had suffered much from headache, confusion of sight, and buzzing noises in the ears. He has had frequent attacks of violent palpitations of the heart. Pulse 74—resp. 44.

A small bleeding was practised; the blood was not inflamed, but the action of the heart was somewhat reduced, the number of pulsations being then only 64, and of the respirations 22.

He was ordered three grains of the digitalis, and this dose was increased to 6, and then to 12 grains, on the two following days; no perceptible effects were produced. Sixteen grains were prescribed; the pulse fell four beats, and the palpitations were less violent. On the following day, a scruple divided into five doses, was taken. The patient vomited his food, and experienced a good deal of nausea; but these symptoms ceased during the evening. Pulse 64. The dose was, however, diminished to 12 grains. During the course of the day, he vomited twice and complained somewhat of vertigo. The pulse was 60, and the number of respirations 28.

*Remarks.* This case illustrates the occasional total inefficacy of digitalis in relieving the symptoms of cardiac disease. It was on the fourth day of the treatment, that any perceptible change on the circulation was first observed, and then this change was very inconsirable.

CASE 3.—*Asthma—Pulmonary Tubercles*—3 *drachms and* 26 *grains in* 12 *days.*

A mechanic, aged 33, was admitted into the La Pitié Hospital on the 15th March, 1833. He complained of a sharp, teasing pain in the region of the sternum, especially when he coughed, or in any way exerted his lungs. On both sides of the chest behind, a sibilous and sonorous râle could be heard; in front, the respiratory murmur was pure, and percussion elicited a healthy sound. The breathing was sometimes exceedingly distressed, and the expectoration of mucus, tinged with streaks of blood, copious. Pulse 96—respiration 28. This patient had experienced several attacks of hæmoptysis, and had for many years been subject to winter coughs. He was bled, and the pulse fell to 68. One grain of powdered digitalis was ordered, and, on the following day, the dose was increased to four grains; the pulse had fallen to 52, but now was 60. Twelve grains ordered. Pulse 46—breathing more free, and also more pure, on auscultation. Sixteen grains ordered; pulse 41—respiration 16. Twenty grs. ordered: pulse 40. On the following three days, the dose of the foxglove was reduced to 16 grains.

There had been occasionally vomiting and considerable nausea; but these symptoms were by no means constant. The tongue presented a natural appearance, and the cerebral functions were undisturbed; the pulse remained at about 40. As the symptoms gradually subsided, the dose of the medicine was again raised to a scruple per diem, and, on the following day, to 30 grains; the patient vomited twice in the course of the night—pulse 40, somewhat irregular in its action. Thirty-six grains ordered; nausea during the whole of the day—pulse 37—respiration 13—cough and expectoration much less. Forty-eight grains ordered; vomiting returned—bowels relaxed—pulse 42, irregular as before. The use of the digitalis was now suspended; the nausea, however, and vomitings continued to recur, the pulse remained at 40, and the respiration at 15—tongue natural—cough quite gone, and the patient left the hospital on the 5th of April, much relieved.

*Remarks.* It will have been observed that, in this patient, the pulse fell on second day of the treatment, when the dose of the digitalis was only two grains; the bowels were then somewhat relaxed, but this relaxation did not increase with the increase of the dose. The stomach began to be disturbed with nausea and vomitings; the pulse fell below 40, and, at the same time, the cough and expectoration abated. These symptoms continued, with little variation, till the completion of the cure. During the whole period, the condition of the tongue remained unaffected, and there never was any epigastric tenderness. Neither the salivary nor urinary secretions were affected.

Case 4.—*Rheumatism—Hypertrophy of the Heart—37 grains in 4 days.*

The patient, a servant, 18 years of age, had for five years, during the Winter months, been troubled with attacks of general rheumatism, and each attack was usually followed by palpitations of the heart. The breathing was always more or less short and distressed; pulse 120, thready—respirations 32—dyspnœa almost constant—the action of the heart sometimes very violent—the dull sound in the cardiac region more extended than natural, and the impulsions of the heart might be heard over every part of the thorax. An incipient bruit de soufflet perceptible; a subcrepitant râle heard on both sides behind.

The patient was bled, and the blood exhibited a partial buffy crust; symptoms not affected—four grains of digitalis ordered. Although the dose was raised first to 9 and then to 12 grains, the action of the heart was not at all abated, and the urinary secretion not increased—indeed, during these two days, a dropsical effusion into the cellular membrane had taken place. The patient was, therefore, bled again, and the blood now presented a stronger crust than before; the digitalis was discontinued. On the following day, there were frequent vomitings and almost constant nausea, and the pulse had fallen to 68, and had become irregular —the respirations were 34. By rest and quiet for a few days, this patient was enabled to leave the hospital.

*Remarks.* The interesting feature of this case is, that the digitalis seemed to be almost quite inert until the second bleeding, when what are deemed its specific effects were first developed.

Our limits prevent us from detailing the particulars of the other cases in which the powdered digitalis was exhibited; we shall, therefore, merely mention their leading features.

In a man, 38 years of age, who had long laboured under pulmonary emphysema, and, at the period of his admission into the hospital, was suffering from acute bronchitis, accompanied with some symptoms of an affection of the heart, venesection and active purgation were employed with decided advantage, the pulse having fallen from 96 to 68. Eight grains of the foxglove were now ordered, and this dose raised, on the following days, to 12, 20, and 25 grains, with the effect of lowering the pulse to 56. Half a drachm was then prescribed, but, as vomitings and nausea supervened, the dose was reduced to 16 grains. Next day the pulse had risen to 72; the quantity of urine was not increased, and, as the head was somewhat confused, the use of the drug was discontinued. On the day after, the pulse fell to 52, and the number of respirations from 24 to 16. This state of amendment continued for several days, and, when the patient left the hospital, the pulse was slow, and the palpitations less violent.

In a case of phthisis, in a state of cavernous ulceration, the digitalis was administered in doses of 6, 12, 16, and 20 grains, on the four days preceding the death of the patient; the pulse, which was at first 128, rose each day, till it arrived at 160.

In another case of phthisis, the digitalis, was taken for four days, in doses of 8, 12, and 16 grains, when it produced considerable disturbance of the gastric functions, and the pulse became somewhat slower; but in spite of these occurrences, the medicine was continued in larger doses, and the vomitings and nausea, instead of being thereby aggravated, actually subsided, and the pulse remained unchanged. On the following day, however, the stomach distress returned, and the use of the drug was abandoned. The pulse had become somewhat irregular, but scarcely abated in frequency.

Such are the results of the experiments in which the powder of the herb was employed; we shall now briefly allude to the results of the exhibition of the aqueous extract.

To a man 38 years of age, who had long suffered from dyspnœa, without any concomitant symptoms of diseased heart, and when his pulse was 72 and his respiration 20, 16 grains were given; on the following day the pulse was 68. The dose increased to a scruple; pulse 60. The dose 36 grains; pulse 54.

Dose the same; pulse 48. The dose the same; pulse 48. Dose 52 grains; and, on the subsequent day, the dose was raised to 72 grains, but the pulse did not fall below 48 beats. Four drachms of the extract were taken in all.

In a case of chronic rheumatism, the aqueous extract was exhibited, although no symptoms of thoracic or cephalic disturbance were present. It was given at first in small doses of only 2 grains, the pulse being at the time 56. The dose was gradually increased to 4, 9, 12, 20, 32, 40, and 48 grains; and the pulse, on the corresponding days, was 64, 60, 60, 56, 56, 56, and irregular, 56, 44, and, again, 52. The stomach was not disturbed until the fifth or sixth day of the employment of the medicine.

The third case is one of acute bronchitis, occurring in a man 58 years of age. He was first bled, and then the aqueous extract was exhibited, in doses of 6, 20, and 30 grains; the pulse fell from 64 to 52.

The three preceding examples appear to indicate that the aqueous extract of digitalis exerts a depressing effect on the circulation; but this is by no means constant or uniform, for three other cases are detailed, in which the pulse was either not affected at all, or actually rose in frequency during the administration of the drug. Two of the patients were phthisical—in the first stage of the disease.

The last case recorded is interesting, and we shall, therefore, give it abridged.

A young man, who exhibited most of the characters of pulmonary tubercular disease, was treated with the aqueous extract. M. Andral commenced with doses of one grain, and gradually raised the dose to 20, and to 36 grains; the pulse fell from 68 to 52, but the digestive, respiratory, and secretory functions were not at all affected. Indeed, the bowels were all the time exceedingly constipated, (the effect of digitalis is usually to induce a certain degree of looseness.) When the dose was raised to 40 grains, the pulse became irregular, and beat only 48; and it fell four when 50 grains were taken. There was no sickness or vomiting all this time, nor any confusion of head, nor giddiness.

The conclusions to be drawn from the preceding data (the authenticity of which is guaranteed by the name of M. Andral), are very different from what medical men would have anticipated; but we suppose that most of our brethren will agree with us in asserting, that the generally received opinions as to the physiological and therapeutic effects of digitalis, have been derived much more from books than from clinical investigations or experiments.—*Archives Générales.*

---

### Influence of Gravity and of a depending Position on the Circulation of the Blood, in Health and in Disease.

To appreciate properly the importance of these influences, it is proper that we attend for a few moments to the condition of the circulation in different parts of the body in its most frequent attitudes and postures; viz. the vertical or upright, and the horizontal. As the former is the most frequently repeated and longest continued, it may therefore be reasonably believed to exert a more influential operation on the current of the blood than the other. Let us consider the effect of the upright position of the body, (and this, we need scarcely say, includes the sitting, as well as the standing posture,) and we shall at once perceive that the arterial circulation in the inferior extremities is thereby facilitated, while the venous circulation is proportionally impeded. It is not therefore surprising that as the body advances in years, the operation of gravity which is acting constantly, except during sleep, against the venous current, should on many occasions induce engorgement of the veins of the leg, giving rise to varices, and to obstinate ulcers. The circumstance of these being almost peculiar to the lower limbs can be explained only on the principle we have stated. The condition of the circulation through the head is the very reverse; the arterial current has to ascend against the gravity of the blood, whereas the venous current downwards is favoured by it. Whenever the upright posture is changed for another, say the horizontal, the circulation is very perceptibly affected; the veins of the face and neck become swollen and livid, the carotids and temporal arteries pulsate with greater force, and headache and confusion of thought are often induced. These phenomena are still more rapidly and more strikingly developed if the head is lower than the rest of the body. From this example we perceive that the veins of the head and neck are nearly passive tubes; their contractile power is very small, no doubt from its being seldom called into play; and hence they become easily distended whenever the current of their blood is not favoured by gravity. The contractile power of the veins of the upper and lower extremities is much greater; but in the case of the latter it is often much weakened by their almost continued state of distention to which they are exposed.

Now the circulation through the other parts of the body also is affected, and that too very materially, by the influence of the gravity of the blood, but in different degrees according to their situations and positions. As a general truth we may assert, that whenever the venous circulation is favoured by the gravity of the blood under ordinary circumstances, *there* will congestions be apt to take place, or to be much increased when they have already taken place, by any change of the accustomed position; and the reason of this is, that such veins have but little contractile power to aid in propelling their contents. To return to the subject of the cephalic circulation, is it not a fact of daily observation, that scarcely any one is able to continue long in a strictly horizontal position? the head must be somewhat raised above the level of the body, else unpleasant feelings come on, which not only prevent sleep but may induce dangerous symptoms. It is not improbable that the less free return of the venous blood from the head when we lie down, may have something to do in the phenomena of sleep. And is it not, in part at least, this cause which keeps up the desire for sleep beyond the requisite period of repose; so that the longer we remain in bed, the longer still we wish to remain? It is not unfrequent to observe in elderly patients who have been, from whatever cause, long confined to bed, a set of nervous and cerebral symptoms supervene, and these may resist every means of relief which may be devised. The perceptive and intellectual faculties become dull and inactive; a state of torpor and apathy, of greater or less degree in different cases, comes on; the patient is unwilling to be troubled with anything, as the answering of questions, and so forth; and when he does return an answer, perhaps it is confused and rambling. These are alarming symptoms, and if they continue and become aggravated we can have no hope of saving our patient.

On dissection of such cases we usually discover some degree of encephalic congestion, and perhaps a trifling effusion within the ventricles. We deem it not improbable that the true source and origin of most of the mischief are to be sought for in the altered state of the cephalic circulation in consequence of the more frequent and longer continued decubitus or position in the horizontal attitude. As it is with the head, so it is with other parts of the body, when they are kept for a length of time in a depending posture. In the chest the stasis of the blood is always more considerable in those parts of the respiratory organs which are lowest; and it has often been remarked, that pneumonia, especially when it attacks those who have been long bedridden, very generally affects the base of the lungs. Perhaps some curious and interesting results might be obtained by endeavouring to ascertain the comparative frequency of pneumonia on the left and on the right side, of engorgements of the liver, and of the spleen, in relation to the ordinary position of the patients during their sleep. It is quite possible that the blood may acquire a tendency to accumulation in particular organs on that side which the person usually assumes while asleep.

In our July No. of last year there is an interesting memoir of M. Piorry, on what he designated "pneumonia hypostatica," or pneumonia arising from a continued state of congestion of certain parts of the lungs, kept up by long confinement in bed. Almost all the cases occurred in old infirm patients admitted into the La Salpetriere, as objects of charity. The mere confinement to bed appeared often to bring on cough and other pectoral symptoms, and these were found to be quite irremediable, if the patients were kept all day in the horizontal position.

Auscultation readily discovered the seat of the pulmonary lesion; the dulness on percussion, and the absence of the respiratory murmur, with the consecutive râles, heard on each side of the spine, shewed that it was the posterior part of the lungs which were chiefly affected; and the post mortem examination confirmed in every case the accuracy of the diagnosis.—[ED.]

The injurious effects of a depending position are well illustrated in the case of the female mamma, when not properly supported, especially during lactation; the veins become much enlarged and distended, and not unfrequently severe darting pains are felt through the organ, giving rise to apprehensions of the commencement of serious disease. Then, too, the very common malady of hæmorrhoids is another striking example of the influence of gravity on the circulation of the blood; and the phenomena of many uterine affections also afford testimony to its operation: thus numerous cases of inflammation of the womb are induced by the patients too soon leaving bed, and getting up; the change from the horizontal to the vertical position favors the more easy flow of blood along the uterine arteries, while it retards the returning current in the veins: hence therefore we may readily explain the occurrence of inflammation or hæmorrhage under such circumstances. Every obstetrical physician knows that it is of paramount importance to enjoin a reclining posture in all affections of the female internal organs of generation.

Again; it is the agency of mere gravity which induces a varicose state of the spermatic veins in men, constituting the diseases of varicocele and cirsocele, and these diseases are invariably aggravated by all causes which are capable of increasing the force of the gravity of the blood, or of relaxing the coats of the bloodvessels, such as exercise, long standing, heat, &c. The use of a well made and well-applied suspensory affords by far the most effectual relief. But the phenomena which result from the influence of gravity are still more apparent and striking in the extremities of the body. If the hand has been long hanging by the side, especially when it is warm at the same time, the veins become full and distended, every minute ramification can be traced, and the whole volume of the soft parts is greatly increased, so that even a feeling of unpleasant tension may be induced: by merely raising the hand and arm, and keeping it for some time in that position, all these appearances vanish, and the member resumes its wonted condition. This affords one of the best examples of the influence of mere gravity on sanguineous accumulations; and we can readily believe that the upper extremities would very often exhibit the effects of such accumulations, were it not for the free and frequent movements of them in all directions:—In the case of the lower limbs, the movements are much more limited, and their position is almost always unfavourable, except during sleep, to the return of the venous blood; whether we are walking, standing, or sitting, the blood has to rise from the feet upwards against the force of its gravity. Hence it is, that varicose distentions of the veins of the foot, leg, and thigh are so frequent, and especially whenever there is any superadded cause, which may impede the easy reflux of the circulating fluid—the pressure of the gravid uterus, of an enlarged ovary, &c. is well known to be a common cause of such a malady. When the larger veins of the extremity have been varicose for some time, and especially if the patient neglects the proper means of relief, the capillary veins become gradually distended and engorged—the surrounding cellular substance becomes inflamed, hardened, and ecchymosed, in consequence of blood oozing out occasionally from the over-distended vessels, and being infiltrated into the cellular parenchyma. It is under these circumstances that the skin not unfrequently gives way, and ulcers, most painful and difficult to heal, become formed. Having thus briefly glanced at some of the most illustrative examples of the influence of gravity, as a cause of inconvenience and disease, we shall now direct the attention of our readers, for a few moments, to certain maladies in which the influence of this agent is conspicuously observed.

In severe cephalic neuralgias, the horizontal position is often found to augment the sufferings of the patient; and the only attitude in which he can find any rest is with his head well elevated. We do not mean to imply that these cases are of an inflammatory nature, yet it is very evident that they are much aggravated by any sanguineous congestion in the parts affected. In phrenitis, otitis, erysipelas of the face, the higher the head is kept raised, the more relief the patient experiences; and when any local inflammation, as of one ear, exists, we uniformly observe that the symptoms are mitigated by lying on the opposite side. Ophthalmia has often been translated from one eye to the other, by the person continuing to lie on the sound side when the inflammation was abating in the other, and this alternation of the seat of the disease may be repeated several times, if the physician's attention be not directed to the real cause. The spreading of erysipelas on the trunk appears to be not unfrequently influenced by the position of the patient; the tendency to spread is generally in a direction to the most depending parts—those on which the patient is resting; and rarely upwards, or to a part more elevated than the spot from which it has started. We have already alluded to the frequency of pneumonic attacks of the lower and back parts of the lungs, in patients who have been long bedridden, from whatever cause; and it is unnecessary to do more than merely again to point to diseases of the rectum, uterus, and male organs of generation, in proof of the influence of position. In the treatment of ulcers of the leg, we are firmly of opinion that repose of the limb, in the horizontal posture, is by far the most important of all therapeutic means; poultices, lotions, and ointments will often all fail; unless this necessary adjunct be attended to at the same time; and even when the patient is not strictly confined, do we not invariably employ what may be termed compensating remedies, viz. strips of adhesive plaster, or rollers from the toes up the whole length of the limb? and the effect of these is well known to be, the taking off the pressure of the superincumbent column of blood from the veins of the foot and leg.

M. Gerdy, about a twelvemonth ago, instituted a number of experiments at the Hôpital St. Louis, on the different methods of treating ulcers; different sets of patients were submitted to the different methods, and each method was employed by itself, in order that the results of each might be justly appreciated. Many of the details have been published in the article "Attitude," in in the Nouveau Dictionnaire de Médécine. We shall mention a few of them.

When the limb on which an ulcer existed was kept upon an ascending inclined plane, it was found that the sore became pale, the suppuration was diminished in quantity, and a crust soon began to be formed upon the surface, and under this the healing went on more or less rapidly. If strips of adhesive plaster were used, at the same time that the elevated inclined position was retained, the cure was still more rapid: it was by combining the elevation with the use of adhesive bandages, and the entire repose of the limb, that the cicatrization of the ulcer was most speedily effected. Several cases of severe contusion were treated on the same plan, with very decided success—the contused limbs being retained in an elevated inclined position during the whole period of the treatment; the decrease of the pain, tension, and tumefaction was sometimes truly remarkable.

M. Gerdy is of opinion, that many white swellings of the joints may be very materially benefited, by an application of the principles which have directed his treatment of ulcers. He recommends that the affected limb be kept perfectly quiet, and on an inclined plane, so that the foot is considerably more elevated than the thigh. He is not yet provided with the reports of any cases to prove the correctness of his ideas; but in one case of elephantiasis of the leg, treated by elevation of the limb, and compression at the same time, the result was most satisfactory—the subsidence of the enlargement was very striking.—*Archives Générales.*

---

### REMARKS ON CERTAIN MONSTROSITIES.

M. Velpeau has appended to a description of a monstrous fœtus some illustrative remarks, from which we shall extract a few of the more interesting.

Eventration, the partial or total escape of the abdominal viscera from their cavity, is one of the most common monstrosities, after those which are connected with a deficiency of part or whole of the cerebral contents. We find examples of this deformity in almost all the scientific collections of the last century, and the journals of the present day very frequently record instances of it. M. Velpeau has himself met with two specimens of this anomaly in fœtuses nearly at the full period, and with numerous others, at earlier periods of gestation. One of the two specimens was a twin fœtus, the other fœtus was born alive and perfectly well formed. The monstrous fœtus was acephalous; all the intestines were fairly out of the abdomen, the parietes of which however were defective only in a very small extent; the anus, sexual organs, and the umbilical cord were normally developed.

In the present case, the deficiency of the abdominal parietes was much more considerable; the whole of the protruded mass being quite naked and uninvested; the skin indeed round the edges of the huge rent or gap presented a natural appearance, and extending from this was a thin pellicle, which became gradually more delicate and fine as it advanced on the viscera, with which it seemed to be confounded.—There was no trace of any external sexual organs nor of umbilicus. The only remains of the cord was an irregular stalk, about two inches long, and which, when it reached the right side of the protruding mass, sent off the umbilical vein to the liver; the two umbilical arteries were much shrivelled; one appeared to be quite impervious, and the other admitted with difficulty a pin's point.

It is an obscure, but very interesting question of embryology, to determine by what means a fœtus, imperfect and deformed though it may be, can be supported and developed, when the umbilical cord is either shrivelled, or, in some cases, is altogether obstructed. Some years ago, M. Velpeau presented to the Philomathic Society a monstrous fœtus, which was quite destitute of any umbilical vessels. Such cases, indeed, are by no means rare, and deserve to be most attentively recorded. Perhaps by comparing an assemblage of such, some pervading analogies of structure might be discovered.—*Journ. Hebdom.*

---

### EXPERIMENTS UPON THE SOUNDS OF THE HEART.

M. Majendie having recently drawn the attention of the Academy of Medicine to the above interesting subject, and propounded certain views of his own, which differ most essentially from those usually received, in ascribing the first sound to the shock or impulsion of the apex of the heart during its diastole against the thoracic parietes, and the second sound to the impulsion of the base of the heart during its systole, Professor Bouillaud, who has for many years distinguished himself by his zeal in the promotion of auscultatory medicine, deemed it proper to have recourse to direct experiments similar to those which Dr. Hope performed on asses.

He laid bare the heart of a strong full-sized cock, having previously satisfied himself by auscultation that its two sounds might be distinctly heard. He then listened to its action at first while enveloped in the pericardium, and then when divested of it; with the naked ear, and with the stethoscope; and not satisfied with one examination, he made several; and the result of these was, that he could always hear quite distinctly the double sound, or tic-tac of the heart, although there was no point of contact between the organ and any part of the thoracic walls. The friction indeed of the heart againt the end of the stethoscope caused a particular sound; but this sound (simply one of rubbing) was so very different from the tic-tac of the organ itself, that it is almost quite impossible that they can ever be mistaken for each other. When the heart was cut out, by being separated from its attachments, it continued to beat for a few moments; but these beats of the empty organ were not accompanied with any perceptible sounds.

The preceding experiment was repeated twice upon rabbits with the same results; viz. the sounds of the heart were most distinctly heard, although neither during its diastole nor during its systole could it come in contact with the thoracic walls.

In conclusion, the Professor states that the results of his direct examination of the sounds of the heart have confirmed him in the opinion that the double bruit or tic-tac, which imitates so closely the clicks of a valve, is, in fact, to be attributed to the play of the valves of the heart.—*Journ. Hebdom.*

---

### Source of Animal Heat.

The chemical doctrine which attributes this important function altogether to the changes which the blood undergoes during respiration is manifestly unsatisfactory; for how can we explain the pyrexial heat of many patients, whose lungs are almost quite wasted away? How shall we account for the sustained temperature of the bodies of persons who have been asphyxiated in irrespirable gases? Does not any sudden fright or terror chill the body? Do we not burn when the rage of anger seizes us? And what makes us shake like a leaf agitated by the wind when an ague-fit is at hand? How is it that any laborious or long-continued exercise of the mind renders us more than usually susceptible to cold? and that the insane, whose minds are, as it were, in a state of abeyance, can often resist a cold which would benumb others who are in health? It is an every-day observation, that palsied limbs, in which the circulation of the blood is as perfect as ever, require external warmth to prevent them being chilled; and in addition to all these proofs of the very immediate dependence, or at least connexion of the process of animal calorification with the condition of the nervous system, it may be stated as a fact of general admission, that the different organs are endowed with heat in proportion to the number of nerves which enter into their contexture.—*Ibid.*

---

### Use of Steel in Menorrhagia.

M. Pigeaux very justly remarks that all uterine hæmorrhages do not depend upon an unhealthy condition of the womb; and that not unfrequently they seem to be connected with a general cachexy of the sanguineous system as their inducing cause. This abnormal state of the blood, which in most constitutions indeed is accompanied with dysmenorrhœa, or even with a complete deficiency of the menstrual flow, gives rise at other times, under circumstances which as yet are not well understood, to profuse and very obstinate discharges from the womb. That these discharges are truly menstrual may be readily ascertained by noticing whether they ever coagulate or not; for it is very generally acknowledged that the true catamenial flux does not separate, as blood is wont to do. By attending, therefore, to this character, we shall be enabled to distinguish between menorrhagia and metrorrhagia: and the distinction is of importance in a therapeutic point of view. No remedy has been found by M. Pigeaux so effectual in counteracting that sanguineous cachexy on which menorrhagia so frequently depends as steel; and the preparation which he prefers is the subcarbonate in doses at first of from 2 to 10 grains, and afterwards of a drachm in the course of the day: when the medicine causes cardialgia, or any irritation of the bowels, he combines it with the subnitrate of bismuth, or with calcined magnesia. The steel should be continued for a month or two after the apparent cure.

The high authority of M. Recamier may be adduced in confirmation of M. Pigeaux' statements.—*Revue Medicale.*

---

### REMARKS ON THE HOOPING COUGH.

M. Blache, in a memoir which lately gained the prize offered by the Medical Society at Lyons, has given a very admirable exposé of this most harassing malady. He very justly considers the hooping-cough as one of the neuroses, and that its seat is most generally in the mucous membrane of the bronchi, and in the extremities of the pneumo-gastric nerves: he alludes to the very frequent association of the nervous disease with bronchitis and pneumonia; but does not permit himself to fall into the error, which is by no means uncommon in the present day, of viewing these complications as essential, or component elements of the malady: it may, and very often does exist, without any trace of them, either during life or upon dissection.

Pertussis, like the other members of the same nosological family, often defies the scalpel of the most practised anatomist to detect its characteristic and pathognomonic lesions; and we are more indebted for any valuable and practical acquaintance which we have of this disease, to the inquiries of the Hippocratic, than of the Anatomical School of Physicians. While we make this assertion, it is not our wish or intention to deny the frequency of the coexistence of inflammatory complications in the course of hooping-cough cases; and we readily admit that much of the mortality of this disease is attributable to neglected pneumonias (of the lobular character) as well as to convulsions and phthisis.

In the treatment of pertussis, M. Blache pays due attention to the three stages of its course; in the first he recommends the same remedies as are known to be best fitted for the relief of bronchitis: in the second, or spasmodic stage, he praises the use of derivatives, emetics, sedatives, and antispasmodics; the most valuable of which he considers to be belladonna: and the third is best counteracted by tonics, such as the lichen, Peruvian bark, sulphureous mineral waters, change of air, &c.

The following sentence of M. Blache's paper is worthy of especial notice.

"In severe pertussis, as in typhus fever, in cholera, and unfortunately in many other diseases, there is another element, besides that of inflammatory action; the nature of the element may be very imperfectly understood; but it is present; and has a most essential influence upon these intercurrent phlegmasias, which may be made to yield at one point, but only to make their appearance at another."—*Ibid.*

---

### MONOMANIA—PHRENOLOGICAL JURISPRUDENCE.

In 1778, a woman at Konigsberg, in Prussia, was found guilty of the dreadful crime of murdering the child of her benefactor, by cutting off its head: and in 1825, at Paris, Henriette Cornier committed the same crime, on a strange child, which had never given any cause of offence to her, and which she had only seen once or twice before.

In both of these cases, the judges were satisfied that the perpetrators were insane at the time of the murders: the first was sent to a lunatic asylum; but the second was condemned at Paris to perpetual labour, and to be branded with a red hot iron on the shoulder!

In the present day, our (the French) magistrates, being better acquainted with the facts on which the doctrine of monomania is founded, are more ready and willing than hitherto they have been, to listen to the system of defence which may be deduced from it. Often (says M. Marc) they are the first to require a medical examination of the culprit with the view of ascertaining the state of his or her mind, in cases wherein formerly not a doubt of the sanity was ever permitted to be held for a moment: and hence it is that in the present day not a few criminal actions are stayed, or altogether superseded by the adoption of such measures as the state of the monomaniac seems to require. This result, so gratifying to humanity, is owing altogether to the exertions of medical men, to whom indeed it is the noblest reward.

In M. Marc's memoir are related several cases of that variety of monomania in which the individual is instigated to attempts on the life or property of others, and yet the mind in other respects appears to remain quite sane. The incendiary monomania has particularly attracted the author's attention; and his remarks and observations are very interesting; but is it quite reasonable to comprehend in this species of insanity the case of a young girl, who, we are told was prompted to the commission of the crime of arson, "less by the effect of an incendiary propensity, than of certain practices and manoeuvres which had been used to disturb her reason, by leading her to believe that she would do a meritorious action, and one agreeable to Heaven, and assuring her of the justness of her actions by the most solemn oaths!"

Thanks to our last revolution (denominated by M. Marc, "a political regeneration,") that the capital punishment which would formerly have been awarded to this poor girl is now commuted to that of perpetual confinement." Those who are interested in medico-legal enquiries, will be repaid by examining M. Marc's memoir in the October number of the *Annales d'Hygiene et Med. Legale.*

### Medical Notanda.

The following short observations and reflections are from the pen of Counsellor Dr. Pitschaft, of Baden, and are inserted in some of the late numbers of Hufeland's Journal of Practical Medicine.

---

### Treatment of Scrofula.

By far the most efficacious remedies against the various forms of scrofulous disease, are, according to the experience of our author, the artificial cinnabar (sulphuret. hydrarg. rub.) conium, cinchona, coffee prepared from acorns, and in obstinate cases, minute doses of the red precipitate of mercury, and the use of salt baths. To children of from one to two years of age, one of the following powders may be given morning and evening:—

℞. Sulphuret. hydrarg. rubri, ℈j.
Herb. cicutæ, gr. ij.
Mercur. precipit. rub. gr. j.
Sacchari alb. q. s.
M. in pulv. xx. divid.

The doses of the medicines are to be increased in proportion to the age of the patients. The use of salt baths, of acorn coffee, and of some of the preparations of Peruvian bark, promote the cure of the disease, especially when the constitutional health is impaired.

Dr. P. urges very strongly upon his readers the great importance of treating scrofula in its early stages, and on its first manifestation by his remedial plan; when the roots of the evil have struck deep, and spread themselves all round, the eradication is necessarily much more precarious. The use of the above powders must be steadily continued for two, three, or four months; and should the stomach be weak and apt to be deranged, a grain or so of the aqueous extract of aloes may be added with great advantage.

---

MEDICINAL PROPERTIES OF ALOES.

In minute doses it is an admirable stomachic, and seems to regulate the due secretion of the gastric juice: that it increases the flow of the bile is unquestionable.

The ancients denominated it "anima ventriculi. Rhazes says "aloe bilem rubeam expellit;" and Aretæus, "aloe ad inferius intestinum bilem ducit." And Ætius has very admirably explained its properties when he writes "Aloe totum quidem corpus non purgat; bilem tamen, qui in stomacho, et ventre, et intestinis fuerit una cum duris excrementis, placide et suaviter educit." To torpor and inactivity of the bowels, especially of the large ones, whether this arises from a deficiency of bile or not, aloes is at once a safe and very efficacious antidote. Dr. P. strongly recommends the following formula, as affording a tonic and aperient pill.

℞. Extract. aloes aquos.

Quininæ sulphatis, āā. ℈j.

M. in pil. xx. One or two at bedtime.

In almost all cases of jaundice, especially of the chronic kind, aloes is a sovereign remedy. The following formula is recommended :—

℞. Extract. aloes liquosi, gr. vi—x.

Extract. taraxaci, ʒij.

Aquæ fœniculi, ℥vj.

Aquæ amygd. amar. conc. ʒj.

M. a table spoonful every hour.

It is very serviceable in all affections of the liver.

RETROVERSIO UTERI—MISTAKE IN DIAGNOSIS.

A woman who had passed through several difficult labours, suffered for three months (during which time the catamenia were absent) from various distressing feelings in the abdomen, and at length from a retention of urine, and a most obstinate constipation; the urine was discharged only by drops; and the use of the catheter was therefore required. For the space of twelve days, no evacuations from the bowels could be procured, although all sorts of purgative medicines had been tried, and the distress of the patient was extreme, from an almost constant tenesmus, complete loss of appetite, nausea, and a tympanitic distention of the belly. Dr. P. was summoned to her relief; he at once suspected the existence of a retroversio uteri; and his suspicions were verified by a vaginal examination. The uterus was easily replaced; anodynes and gentle aperients soon restored her to perfect health. Six weeks afterwards she had a discharge of a large black mass from the vagina, but no traces of a genuine mole could be detected in it.

EPISTAXIS AND HÆMOPTYSIS.

The application of cold washes to the testicles to arrest hæmorrhages from the nose and chest has of late years been too much neglected.

In females the cold may be very conveniently applied to the breasts; and the most easy and effectual method is, to lay a bladder filled with pieces of ice on the mammæ.

In violent hæmatemesis the cold may be advantageously applied to the throat. The operation is probably exerted on the par vagum; and Dr. P has with the same remedy sometimes succeeded in arresting an obstinate vomiting.

SYMPATHY BETWEEN THE CEREBELLUM AND THE GENERATIVE ORGANS.

It is well known that Dr. Gall considered the cerebellum as the organ of the sexual passion. Plato says that the semen comes from the spinal marrow. In Meckel's Archives of Physiology for 1823, are detailed the particulars of the case of a two-year boy, in whom a premature development of the generative organs, and of the occipital region was contemporaneous. Beheaded and hanged criminals exhibit erections and pollutions; and Dr. Otto has communicated a most interesting account of the appearances of the generative organs in a woman who was hanged.—(Vide his Rare Observations in Anatomy.)

M. Serres has detailed some cases in which constant erections of the penis were among the symptoms present in chronic inflammation and congestion of the cerebellum; and in the 4th volume of Majendie's Journal, we are told, that if the cerebellum of some animals be exposed, and a stilet be forced into it, the penis becomes stiff and erect, and that if the stilet be pushed down the spinal canal as far as the lumbar region, seminal emissions take place.

Such are some announced physiological facts; and the consideration of them may lead us to a more successful system of therapeutics in cases of morbid excitement of the sexual functions.

Among the internal remedies which have acquired the name of anti-aphrodisiac, the least problematical is camphor. According to Fodéré's experiments on the operation of various drugs it appears that camphor acts specially on the cerebellum.—(Vide Archiv. Générales, tom. 3.) And Drs. Duncan, Perfect, and Osiander have strongly recommended its use in that form of melancholy and mental disturbance, which not unfrequently occurs during the period when the sexual feelings are developed. A more important remedy is the application of cold to the occiput and nape of the neck. Frequent ablution with cold water is the simplest and most effectual method: occasionally, too, the local detraction of blood by leeches, or the cupping-glasses, may be of great service. Our attention ought to be directed to this treatment in cases of epilepsy accompanied with priapism and pollutions; and we should remember that it is in no way improbable that the same part of the nervous centre may be indisposed in many examples of hysteria; in this disease it is by no means uncommon for the patient to complain of an uneasy feeling in the back of the head and neck; and the phenomena of catalepsy must assuredly be somehow dependent upon an affection of the spinal marrow.

Baglivi long ago observed—

"Frequenti experientia constat, ex affectione uteri dolores verticem et occiput precipue invadere; pariter mulieres hystericis obnoxiæ affectibus sensum quemdam frigoris in vertice capitis habent; estque hoc precipuum hysteriæ diagnosticum."

---

### Use of Pomegranate Bark against Tapeworm.

Of late years many authors have strongly recommended the employment of this remedy for expelling different kinds of the intestinal worms:

"Multa renascentia, quæ jam cecidere."

Frequent allusions are made to the therapeutic qualities of the pomegranate in the ancient authors. Pliny, in his Natural history says, "Radix decocta succum emittit, qui tænias necat;" and Dioscorides, "Radicum decoctum latas ventris tænias potu pellit et enecat." Celsus directs, "Cum pridie multum allium ederit, vomat posteroque die, mali Punici tenues radiculas colligat quantum manu comprehendet; eas contusas in aquæ tribus sextariis decoquat, donec tertia pars supersit; huic adjiciat nitri paulum, et jejunus bibat;" and lastly Avicenna, "Cortex granati cum vino extrahit vermes et ascarides, et assumitur cum sua dispositione, aut sumitur ejus decoctio."

---

### Effects of Mercury on Living Bodies.

From the experiments instituted by Caspard (Majendie's Journal of Physiology) it appears that the development of the embryo in the eggs of birds, of the amphibia, snails and insects is arrested and destroyed by the vapour or exhalations of this metal. Consonant with these results, is the fact vouched for by Van Helmont, Hoffmann, Baglivi, and Bremser, that water in which quicksilver has been boiled, possesses anthelmintic properties; and the observations of Kluge prove, that calomel given to a pregnant woman exerts injurious effects upon her fœtus, whereas other preparations of the metal, in which the oxidation is more complete, do not so. Baglivi, in the following sentence, alludes to the anthelmintic properties. "℞. Mercurii crudi recte purgati, unc. j. Aquæ graminis et portulacæ, ana, uncias quatuor; macerentur per duas horas sæpe ac fortiter agitando; postea decanta aquam et cola, relicto in vase mercurio. Non datur prestantius pro fugandis vermibus hoc remedio, ut observavimus, docente Georgio Bateo, Angliæ archiatro."

The odour of other metals, although scarcely appreciable by us, seems to be disagreeable to many of the lower animals. It has been remarked, that a dog, even though well trained, is reluctant to seize and carry a piece of gold; and perhaps the mere effluvia of tin, when given as anthelmintic, may have some pernicious effects on intestinal worms.

---

### EXAMPLES OF SYMPATHY IN DISEASE.

A very frequent, but hitherto almost unnoticed symptom of diseased liver, is a feeling of irritation and pressure on the larynx and pharynx. Dr. Pitshaft, when he first announced this, was not aware that former writers had noticed it; he finds however in Baglivi's work, the following sentence. "Jecore affecto dolores ad jugulum e directo fiunt."

The etiology of this symptom is probably to be sought for in the distribution of the par vagum; and in the same manner perhaps we may explain the supervention of aphonia on many occasions. How common this is when the mind is much agitated; terror, rage, and immoderate desire are well known to rob the persons of their speech.

> Illi membra novus solvit formidine torpor,

> Arrectæque horrore comæ, et vox faucibus hæsit.

Valerius Maximus mentions that Ægles Samius, a wrestler, who was quite dumb, when unjustly deprived of the reward of his victory, recovered his speech in the heat of indignation. It is to be kept in mind, that the liver and also the spleen, are always more or less affected after a paroxysm of mental agitation. Isodorus ingeniously remarks, "splene ridemus, felli irascimur, corde sapimus, jecore amamus;" and another author expresses the same sentiments, when he writes, "cor sapit, pulmo loquitur, fel continet iras, splen ridere facit, cogit amare jecur."

---

### USE OF MERCURY IN RHEUMATISM.

Dr. Burdach has lately published several cases in confirmation of the good effects of small doses of the corrosive sublimate in cases of rheumatism. For the last twenty years Dr. Pitschaft has been in the habit of employing mercury against this disease; the preparation which he prefers is the red precipitate; it is more mild, and quite as efficacious as the sublimate. He gives it in doses of from one-eighth to a fourth part of a grain twice a day; should it irritate the alimentary canal, a small quantity of opium should be combined with it. When the periosteum is affected, the sabina will be found a useful adjunct; when the nervous system is irritable, the chenopodium may be given, and when the lymphatic system is torpid, the arnica and calamus may be given along with it. As an external application to the affected parts, Dr. P. recommends a salve prepared with caustic ammonia, or one with borax, if there should be any œdematous swelling of the limb.

---

### TREATMENT OF EPILEPSY.

In almost all cases of epilepsy, depending upon some disturbance of the cerebral circulation, the following powders will, if persevered in for a considerable time, mitigate if not altogether cure the malady.

℞. Cinnabar. fact.

Magist. *wismuth.* (qy. b.)

Herb. nicotian. āā ℈j.
Extr. aloes aquos, gr. v.
M. in pulv. xx. divid. One twice a day.

Many of the older authors had great faith in the virtues of cinnabar, and it acquired the name of magnes epilepsiæ. Dr. Pitschaft regards it as a 'remedium divinum.' We know that the Eastern nations very generally employ it, combined with musk in cases of hydrophobia.—*Hufeland's Journal.*

---

### Extracts from the Case-book of Dr. Mombert.

RUPTURE OF THE UTERUS; GREAT INEQUALITY OF THE THICKNESS OF ITS PARIETES.

Dr. M. was summoned to a woman who had been in labour for several days, under the care of a midwife, but before he could reach her abode she had expired. On examination per vaginam, the os uteri was dilated to the size of a dollar, and the head of a child could be distinctly felt. Dr. M. proceeded at once to extract the child by the cæsarian operation. As soon as the abdominal parietes were divided, an immense mass of blood was discharged in a profuse stream, and the floor was deluged with the gush; the intestines protruded out of the wound, and incommoded the steps of the operation; when these were replaced, Dr. M. very naturally expected to find the distended uterus, but not so: on continuing the examination, he found the fœtus, with the placenta attached, and surrounded with much fluid and clotted blood, lying in the cavity of the pelvis, behind the ruptured uterus. The child was quite dead. The uterus presented a very remarkable inequality in the thickness of its walls. The rupture was on the posterior surface, extending from the cervix to the very fundus, and here the substance of the uterus was not thicker than pasteboard; the breadth of this exceedingly thin portion was about an inch and a half. At the fore and upper part of the fundus, and in some parts of the sides, the uterine parietes were from three and a half to four inches thick; and it was observed that they became gradually more and more attenuated towards the posterior surface. The texture of the uterus throughout was unusually pale, but possessed the ordinary firmness of consistence.

The patient was about 30 years of age, and had borne several children. In the present labour, the pains at first were strong and severe, but became weaker and weaker. All of a sudden the alarming symptoms which indicate rupture of the womb, such as the feeling as if something within had given way, sharp fixed pains through the pelvis, syncope &c. set in; but her ignorant attendant had no idea of the fatal calamity. It is to be observed, that for some hours before death, the labour pains had utterly ceased.

*Remarks.* Several similar cases are recorded in Voigtel's Pathological Anatomy. In one example, the uterine walls at the site of the rupture were not thicker than three sheets of writing paper folded together, and had a blueish appearance. In another, the reporter says, that they did not exceed the thickness of the back of a knife, whereas at the fundus they were at least two inches thick.

---

LUMBRICUS FOUND IN THE WOUND AFTER AN OPERATION FOR HERNIA.

The patient was a youth, 18 years of age, and laboured under a scrotal rupture; it had become irreducible after a scuffle, and could not be replaced by the taxis. He would not consent to the operation till the sixth day after the incarceration. The prognosis therefore was not a very favourable one. On dividing the abdominal parietes and the peritoneum, the omentum presented itself; it was of a brownish black colour, and quite sphacelated; and the noose of gut contained in the sac was of a dark colour, but it did not feel soft and lacerable to the fingers. With much difficulty the bowel was replaced, and a considerable portion of the mortified omentum was detached.

The future progress of this case was much more favourable than could have been anticipated; the constitutional symptoms became favourable, and the wound shewed a disposition to heal, although the purulent discharge from it had never a healthy appearance. On the thirteenth day after the operation Dr. M. was suddenly summoned to his patient, whom he found in the greatest consternation, in consequence of his having found in the wound when he was dressing it, three large lumbrici, which he by gentle pulling had extracted—they were each about six inches long. Dr. M. at first suspected that the worms might have been discharged from the anus, and afterwards have crept up to the wound, and there lodged themselves; but as this had been covered and secured, not only by cerate dressing, but also by adhesive plaster and bandages, the idea was quite improbable. From the hour of the expulsion, the wound assumed a much more healthy aspect, and in eight days afterwards it had entirely healed. Dr. M. states that he is assured in his own mind that the bowel was not at all cut or injured during the operation.

*Remarks.* The occurrence of worms in cases like the preceding has been noticed by several authors, as Haller, Schulze, Schmucker, &c. and lately a very interesting example was published in the July number of this (Hufeland's) Journal.

---

### SIMPLE TREATMENT OF A FACIAL NEURALGIA.

Dr. M. was called to the relief of a lady 42 years of age, who had for some time been suffering from severe infra-orbital neuralgia. As the attacks of pain were periodic, returning generally every morning, it was suspected that a masked intermittent fever might be the exciting cause of the malady; [why was this idea not acted upon and the quinine &c. not exhibited?—*Rev.*] In vain opium, belladonna, conium, laurel water, and many other remedies were tried —they afforded only a temporary relief. Dr. M. happening to be with his patient during a very severe paroxysm, put in trial an expedient which, it had frequently occurred to him, might be of use: he directed a small stream of ice-cold water, by means of a clyster pipe, upon the pained part; the effect was instantaneous and most soothing; but soon the pains returned as acute as before; he now applied compresses wrung out of hot water, and on removing these, he again made the stream of cold water play upon the part; the relief was immediate. This treatment, the alternate application of heat and cold, was repeated as often as the pain returned; each paroxysm was less severe than the preceding one, and after a few days the disease was entirely checked. Four weeks afterwards the patient was safely delivered of an infant, and from that time, six years ago, up to the present period, she has remained free from her torturer.

[It is to be remembered that the preceding was a case of puerperal neuralgia.—*Rev.*]

The second case occurred in the person of a young lady, 22 years of age, who had been afflicted with a similar facial neuralgia for three months, and who had been subjected to a great variety of remedies, without any avail. Dr. M. recommended the trial of the alternate application of a high heat and cold; and the result was most gratifying. Every paroxysm could be arrested by this means; and ultimately a perfect cure was obtained, accelerated no doubt by the employment for some time of sulphureous baths.

---

### RESULTS OF DR. M.'S EXPERIENCE OF IODINE IN SCROFULA.

It appears that bronchocele and other forms of scrofula are very common in the district [Wanfried] where the Doctor is settled. The town, of the same name, is situated in a valley surrounded with lofty calcareous mountains. The water which is drunk is good, although hard, in consequence of the quantity of carbonate of lime which it contains; but the general condition of the poorer classes is deplorably wretched; their habitations being damp and unwholesome, and their food and clothing scanty and miserable. Dr. M. has remarked, that strangers who come to reside in the district generally remain free from the malady, but that their children are almost quite as much exposed to it as the indigenous inhabitants. Many children are born with it; sometimes it disappears of its own accord within the twelvemonth, but it is apt to return in the second or third year. The use of the hydriodate of potass ointment (a scruple to the ounce of lard) is generally sufficient to disperse the swelling in such cases, and the internal exhibition of iodine, or of its preparations, is unnecessary. Dr. M. has never in his practice had occasion to remark any injurious effects from the internal use of the tincture of iodine, in adults. Once indeed he saw the mamma of a young female very much reduced in size, while she was taking the medicine; but by discontinuing it for a time, and giving fennel tea freely the organ regained its dimensions. In this case the iodine had caused a considerable increase in the flow of the catamenia. The burnt sponge seemed sometimes to act more beneficially than the iodine, and vice versâ. It is therefore a good practice to alternate the use of the two remedies. The ung. hydriod. pot. should be used at the same time. This treatment must be cautiously watched in plethoric and irritable constitutions, as hæmoptysis is an occasional effect of it. It appears that a good many of the cases of asthma which present themselves to Dr. M.'s notice, are connected with scrofulous enlargement of some of the intra-thoracic glands; thus, in one example, he found on dissection that the trachea and even the œsophagus were compressed by a tumor which occupied the site of the thyroid gland.—*Hufeland's Journal.*

---

### Case of Somnolency which continued for four Months.

When Dr. Oelze, the narrator of this interesting case first saw the patient, a girl eleven years of age, she had been asleep for about six weeks. He visited her on the 18th of May, 1826, and learned the following particulars from the attendants.

At the beginning of the year she had been attacked with measles, which were prevalent at that time in the village; the disease was mild, and her recovery rapid: but in a few days, after she had been able to leave her bed, she complained much of a severe pain in the right ear; this continued for about eight days, and then subsided into the feeling of a dull ringing sound, so constant and so distressing, that the little patient had no sleep for nearly five weeks; otherwise there was no indisposition, except general weakness. Soon afterwards however violent headaches came on; and these were succeeded by colicky pains, which were always increased after eating. The pains in the abdomen ceased in the course of a fortnight, and then for eight days she was distressed with pains in all the limbs and joints of her body. On the 3d of April she fell into a state of torpor or sleep, from which no means which had been tried could rouse her, and she had continued so until the day when Dr. O. first saw her. Some days she uttered a moaning sound once or twice; but with the exception of this, she had lain without food, and as still as a corpse, till two days before Dr. O. had visited her; when, as the patient made an effort to weep, her mother had introduced a small quantity of milk into her mouth. On the following day a similar attempt at weeping was repeated, and this time she was made to swallow half a cup full of coffee. She occasionally coughed, and now and then turned herself in the bed. No urine had been passed since the commencement of the somnolency, and only once a very small quantity of hardened fæces had been discharged. The skin had all along been unusually dry.

Dr. O. describes her appearance as follows. The face was pale, the body generally somewhat emaciated; the chest was well expanded, the abdomen drawn in, and the skin of every part felt exceedingly dry to the finger. The respirations were unusually short, but so gentle was each act, as to be with difficulty recognizable: they were however not uniform in this respect; for sometimes the chest appeared to heave up more considerably, although this did not seem to be the result of a deeper breathing; while at other times its motions could not be perceived. The pulsations of the heart were also very irregular in their frequency and strength: usually after each stronger beat numerous smaller ones followed; then the heart seemed to rest a while quite quiet, or only to be affected with a slight tremor: the pulse at the wrist however was tranquil and much more regular, but very small and easily compressible: it beat from 84 to 94 in the minute. The hands were kept always firmly contracted. When strong hartshorn was held to the nostrils, and applied on the upper lip, the patient sneezed and rubbed her nose with her hand. The effects of galvanism were then tried; slight shocks were sent sometimes from the pit of the stomach to the forehead, eyelids, ears, &c. and at other times from the soles of the feet to the head, arms, and so forth. At first these shocks were evidently distressing, and caused her to draw herself, as it were, together, and to begin crying; the ears appeared to be the most sensitive parts to the galvanic irritation, for whenever one of the poles was applied to them she endeavoured to cover them with her hands, and strove to say that they were pricked and very painful. When asked if she felt pain in any part, she pointed to the neck. Dr. O. told her he wished her to take some coffee, and she permitted a spoonful or two to be poured with a spoon into her mouth; but the efforts to swallow it were evidently most painful and difficult, and it was sometime before all of it disappeared. The deglutition was easier after both sides of the neck, along the track of the œsophagus, were well galvanized. When the hands were galvanized, they were more contracted than before, and never became properly extended. It was remarked that no particular twitchings of any muscles were ever induced by the application of galvanism. The patient seemed to retain perfect consciousness, and to observe whatever was going on around her, although the eyes were kept always closed. When asked to look at any thing, it was easily seen that she strove to do it; but the eyelids were never more than very imperfectly separated from each other. When drawn asunder a slight resistance was felt, sufficient to indicate that a state of spasmodic contraction of the orbicularis had existed; the eyes had a staring look, and rolled slowly from one side to the other—the pupils were considerably dilated. When the patient uttered any cry, it was observed that she took long and deep inspirations without any apparent impediment or difficulty; but no sooner was she asleep than the breathing became again short and much quickened. It was curious to remark how quickly and almost instantaneously she relapsed into sleep, even after the excitement which had been produced by galvanism was so violent that she had been screaming, kicking with her feet, and beating with her hands against all attempts to repeat it. However much agitated the rest of the body was, the eyes were always kept shut.

Dr. O. having persevered in the employment of galvanism for a full hour without any satisfactory good effects, discontinued it; and concluding from the history of the case, that the "primum mobile," or at least one of the most prominent features of the case, was the cessation of the cutaneous secretion, directed his efforts chiefly to restoring this to its normal condition. For this purpose he ordered a mixture of infusion of valerian, camphor, and the compound sulphuric spirit.

*From the 10th to the 30th of May.*—It is stated that the child slept very tranquilly after the application of the galvanism until the next morning, when she seemed rather more lively, and told her mother, that the ears, chest, and abdomen in particular, had been very painful from it. From that time a cupful of milk was given, by spoonfuls, each day, but it had not agreed with the stomach, for indigestion, nausea, and even vomiting had been almost always brought on, after it was given. The medicine had caused nearly the same effects. Since Dr. O.'s first visit the sleep had not been so constant, nor yet so profound; for the mother had been able to obtain a few answers from her every day. At times a gentle perspiration had bedewed the surface. On the 27th of this month, for the first time since the third day of the preceding one, some urine was voided, and the patient had indicated by signs that she wished to be taken out of bed, in which two or three hardened scybalous masses were found.

Dr. O. visited her on the 30th—she was asleep, and the respirations were so gentle that he could scarcely perceive that she breathed at all. The same irregularity in the movements of the chest were noticed, as on the first report; and the actions of the heart were still as confused; but the pulse retained its former regularity, and now beat 74 in the minute. The hands were in the same contracted state, and when Dr. O. inserted his fingers between those of the patient and the palm of her hand, he found that they were so very firmly grasped, that it required a considerable effort to withdraw them. She took no notice of what was addressed to her; and even shaking her body and limbs seemed not to affect her. Ammonia held to the nostrils, produced only a slight twitching of the features.

The application of galvanism was again resorted to, and similar effects were obtained from its employment. It seemed this time that the patient remembered the annoyance which she had experienced before; for she struggled very violently against every attempt made to repeat the operation. The transitions from agitation to sleep were now not so rapid as on the former occasion. Deglutition was quite as difficult as before; and most of the other phenomena were unaltered.

A warm bath, in which a considerable quantity of common salt had been dissolved, was ordered for her. When put into it she attempted at first to support herself with her hands. Some frothy saliva escaped from the mouth, and she then lay still and motionless as a corpse; the hands became more compressed, and indeed altogether the spasmodic state or tendency of the body was rather increased than diminished by the remedy; but upon being removed from the bath and wrapped in warm blankets, a strong perspiration broke out, and this lasted for upwards of two hours. The medicines which Dr. O. prescribed at this visit were the following; a grain and a half of the flowers of zinc every night and morning, and an enema of tobacco and chamomile infusions once a day.

*From 30th of May to 9th June.*—The sweating, which had followed the use of the warm bath had retured although less profusely, several times afterwards, but with no amendment of the symptoms; indeed the stupor seemed to have increased somewhat; for the patient had spoken scarcely a word, and not a mouthful of nourishment had been taken. Only two injections had been given, and these had never returned; it was therefore deemed unsafe to repeat them. A sort of general spasm, or convulsive fit, (of a truly opisthotonic character) had taken place almost every day, and this was always preceded and announced by the patient's screaming. The spine and head were violently bent backwards, and the limbs rigidly extended. This state continued for three, four or five minutes, and then the spasm abated, and the joints became more flexible. A small quantity of urine was voided on the 8th of June.

The next day Dr. O. saw her, and found her very nearly in the same condition as at his former two visits. The breathing and circulation were unaltered; the alæ nasi were observed to dilate and close more powerfully, and the eyelids frequently to be affected with tremulous movements. The galvanism was repeated, as far as the patient's struggles permitted its employment: it had the effect however of forcing her to answer all questions more readily and distinctly. Soon afterwards several paroxysms of spasm came on, at intervals of from eight to twelve minutes. One grain of calomel with one of the red sulphuret of antimony and three of sulphur were ordered to be given morning and evening, and an enema composed of valerian, tobacco and chamomile infusions.

On the 16th it was found that the attacks of cramp had been less frequent on the whole, although there were generally still four or six daily. The patient had drunk a little milk now and then, but she had not spoken at all; and only once was any urine discharged. The pulse beat from 75 to 80; the other phenomena unchanged. The effects of opium were now tried; fifteen drops of laudanum were given, and repeated every hour for three times. When the second dose was given she became more lively, and began to scream out; thereupon a paroxysm of opisthotonos came on, and lasted for several minutes. A warm bran bath was ordered, and the same injurious effects followed as after the use of the water bath. The powders prescribed at the former visit were continued.

*July 4th.* Very little change in any of the symptoms; once only there had been a discharge of urine, and of fæculent scybala; and of late the paroxysms of cramp had become more frequent; some days they recurred almost every hour, and their duration also was longer than before. The calomel, &c. powders had been given as often as possible; for frequently not a grain or drop of any thing could be got down; and it was only to be effected when the mouth was opened during the crying which preceded the paroxysm. At other times it was kept firmly closed. Numerous clysters had been given, and every one of them retained. The surface of the body was almost always cold, although the temperature of the air was at this time very high.

About a week after this date the child began to be less drowsy, and to notice more and more whatever was going on in the room where she was. It was quite apparent that she understood the conversation of those who were talking to her mother; and on one occasion, she intimated that she wished her chest to be rubbed again with a hartshorn liniment, which she had observed to have been put aside in a cupboard. She took more willingly small quantities of milk and of coffee; but both of these drinks were generally vomited soon after being swallowed; this irritability of the stomach had existed from the commencement of her malady. Urine had been voided once, in the last ten days.

*25th July.* There had been some abatement of the somnolency since last report, and the attacks of cramp had been less frequent and severe. She had taken more nourishment, although vomiting still occasionally followed the effort of swallowing. The sensibility and consciousness were also more distinct; but though observant of what was done or said in her presence, she had not of late spoken at all, and had answered questions put to her only by winks and nods.

The urine had been discharged three times, and the bowels relieved twice.

The action of the heart was less irregular than hitherto, and the movements of the chest were more uniform; the eyelids were more readily separable, and frequently agitated with a tremulous motion; the pupils were now natural. When Dr. O. visited her she was in a profound sleep, which was not in the least disturbed by shaking her violently from side to side.

The powders and infriction on the spine were ordered to be continued.

From this date the somnolency became very much less, and the tetanic convulsions almost quite ceased.

It had been observed that the degree of the former had been always proportionate to the violence of the latter; and now both symptoms abated simultaneously. The appetite gradually returned, although the stomach continued to reject part of the food; the bowels and urinary bladder relieved themselves daily. The eyes were observed to be very sensitive to the impression of light.

By the middle of August she had recovered so far that she remained lively during the whole of the day. When she attempted to walk, her gait was awkward and tottering; the spine appeared to be exceedingly weak, and, upon any exertion, the breathing became oppressed and the circulation much hurried. The speech, however, was still almost quite deficient, and when she was asked the reason of it, she pointed to her chest, where, it seemed, she felt much embarrassment and distress. However, in the course of another fortnight, it was greatly restored, and from this date the patient was pronounced to be quite well. The child, it appears, had no remembrance of her illness, and of what had been done to her during its continuance.

*Reflections.* The somnolency, in the preceding case, seems to have been induced by the mismanagement of an attack of measles, especially during the eruptive stage. The early symptoms were those of pain, and feeling of noises in the right ear: as these subsided, severe headaches, then pains in the abdomen and limbs, set in, and, lastly, the somnolency and tetanic spasms succeeded. This succession of symptoms seems to indicate the operation of a rheumatic-gouty diathesis as the cause of the malady.

The physiology of such a case is certainly obscure:—As noticed before, the co-existence and co-abatement of the drowsiness and convulsions were probably owing to the same morbid state; but what the nature of that state was, it is not easy to determine; perhaps some peculiar pressure on the nervous centres. It is not a little singular, that the organic functions can be maintained for such a length of time, without any fresh supply of nutriment, as they were in the present case. For six entire weeks, not one mouthful of food was swallowed; and, for the next five or six, only from a half to one small cupful of milk and coffee in the course of the day; and half of these quantities was generally rejected by vomiting.

The respiratory function was, indeed, imperfectly performed, and the circulation was more or less disturbed—so also were the secretions. But yet the emaciation which took place was by no means very striking, and nothing like what might have been anticipated from so lengthened an abstinence. A feature of the case worthy of notice is, that the arterial pulse was steadily regular, although the actions of the heart were almost always irregular and confused. This would seem to indicate that, in some convulsive diseases, the actions of the heart and arteries are independent of each other to a certain extent. As to the therapeutic deductions, Dr. O. is inclined to attribute most of the benefit from the medicines employed, to the mercurial preparations; and, of these, the most efficient seemed to be the calomel.

The venerable Hufeland has appended a remark or two of his own to the preceding report of Dr. O. He alludes to the metastatic origin of the somnolency and cramps, and conjectures that a certain degree of cerebral effusion had existed during the greater part of the existence of the malady. He attributes the recovery of the patient to the agency of the mercury having excited the action of the absorbents. He approves of repeated blisters, applied to the nape of the neck.—*Journ. der Pract. Heilk.*

---

### Use of Aconite in Neuralgias—and of Creosote in Ulcerations.

M. Roche has of late used the extract of aconite in doses of one grain, to be gradually increased, with very considerable success, against facial neuralgia and other nervous pains. He gives it sometimes alone, and at other times in union with opium. M. Tealier has followed the practice, and reports most favourably of it. The cases, however, which he has adduced were all of them of recent standing; and it is well known at the commencement of the malady, a complete cure may very generally be effected with other means.

M. T. has also communicated the details of two cases of painful ulceration, in which he had used externally the creosote (a short history of this newly-discovered substance will be found in our last No.) with pleasing effects. The first was one of cancerous ulceration, the pain of which was most agonising to the poor patient. M. T. applied rags, wet in a dilute solution of the creosote, to the sore. It caused at first very severe burning pain, which extended from the mammæ all round, and lasted for upwards of an hour; it then ceased, and the patient was easy for the next ten. The application was afterwards repeated, and always with temporary advantage.

The second case in which the creosote was employed was that of a female, 34 years of age, and mother of a large family, who was labouring under chronic metritis and ulceration of the os tincæ.

One part was mixed with three of water, and a plug of charpie, wet with the mixture, was introduced into the vagina, and the mouth of the womb freely wetted with it. The pain which ensued was quite distracting; injections of cold water were employed, with partial relief—but the suffering did not altogether cease until the 2d day. The patient then, however, had experienced so much benefit, that she was able to rise from bed (to which she had been confined for two months); and when the os uteri was examined some days afterwards by the speculum, it was found that the appearance of the ulceration was much improved.

The application, but in a much more diluted form, was renewed several times; and the eventual result was most gratifying and completely successful.—*Révue Médicale.*

---

### DEATH CAUSED BY CYANURET OF POTASS.

To a patient, labouring under severe neuralgia of the trunk (abdomen?) a lavement, in which were dissolved six grains of the cyanuret in a moist state, was given; and, as it did not afford relief, it was twice repeated afterwards. Each time, strong convulsions and violent contractions of the limbs were induced—the eyes became fixed, and the pupils were dilated. The patient was, however so much benefited, that he was able to rise from bed, to which he had been confined for a twelvemonth.

A fourth injection was given, without any marked effect, and a fifth 36 hours afterwards; the same dose was exhibited, but the cyanuret had been taken from a quantity which had become dry, from being kept in a closed bottle, and which was almost quite inodorous. Soon after it was received, severe convulsions set in, the respiration became slow and embarrassed, the limbs were cold, and death ensued in the course of an hour.

M. Trouve, the reporter of the case, attributes the fatal accident to the greater activity of the cyanuret when it is in a dry, than when it is in a moist state. —*Ibid.*

---

### ON THE FORMATION AND ARRANGEMENT OF THE EPIPLOONS IN THE FŒTUS.

According to the celebrated anatomist Meckel (whose death science has had lately to deplore), there are no traces of the epiploon in the very early periods of fœtal life. At the end of the second month, it appears as a projection at the great curvature of the stomach; this projecting part gradually becomes lengthened, and chiefly its lower portion; but it is not yet in contact with the transverse colon. While the mesentery is forming, the epiploon descends a little more—its inferior layer it joined to the mesocolon, and is soon confounded with it; this adherence exists in the fourth month—but it is still so loose, that we find no difficulty in separating the epiploon from the stomach There is not the slightest appearance of fat, at first, between the layers.

Meckel and Kieser had previously demonstrated that, 1, The stomach is placed, during the fourth and fifth weeks of intra-uterine life, nearly quite perpendicularly; and that, at these periods, it is only a semicircular expansion ("renflement") of the intestinal canal —2, This canal proceeds directly to the umbilical cord, then turns back at a sharp angle into the abdomen, till it reaches the anus. In this state, the stomach and intestine are attached to the posterior wall of the abdomen by means of a vertical fold, which, coming from the spine, is directed to the great curvature of the stomach, where it becomes separated into two layers, of which the left one passes in front of the anterior surface of this viscus, and the other, or right one, passes behind; these layers then meet each other at the smaller curvature, and proceed on to invest the liver. This fold is, therefore, truly a mesentery to the stomach, and Professor Muller denominates it mesogastrium.

In proportion as the position of the stomach becomes more horizontal, and the pylorus to be directed towards the right side, the mesogastrium acquires an oblique direction from left to right, and the layers become slightly plaited, and lengthen out from the point at which they are united, viz. at the larger curvature.—*Meckel's Archives de Physiol.*

---

### Fatal Blow to Homoiopathism in Russia.

The celebrated Berlin surgeon, Dieffenbach, has very truly said, that this new doctrine offers a vast field to the most absurd reveries and antiquated superstitions, which some shallow men always prefer to whatever is clear and palpable; and all who have perused their oracular book or institute, the "Organon of Medical Science," as it is modestly called by the author, must have arrived at the same conclusion. There is an utter destitution of all rational proofs of the opinions, or rather dogmas, there announced. Proceeding from a few appearances, at best of very exceptionable accuracy, Hahnemann leaps at once to the assertion—that no medicine ever cures a disease, unless it is capable of exciting in a healthy system symptoms altogether analogous to those which it is employed to relieve. Almost all chronic diseases, we are informed, are caused by ill-cured itch! There is no proof given of this—only we are told that it is so!!! Then another wondrous discovery is, that the ten-millionth part of a grain of charcoal is a very active agent in some diseases; and that many medicines are extraordinarily exalted in efficacy, by the number of times the vial which contains them in solution is shaken!!! Yet, such is the hungry credulity of mankind, that all these good things are swallowed, nay, even digested, if we may judge from the products, we mean of the brain, which, with all suitable consistency, are inversely proportionate to the ingesta. Born and nursed in Germany, that fatherland of wild phantasies, this curious doctrine has been diffused over Switzerland and Italy—has entered France, and reached Lyons, and even Paris. [It has found a home in our own country also.—Rev.] Like every novelty, it blinds and infatuates many for a time. When we hear of old-established practitioners, not to allude to numerous young men, who cannot possibly be influenced in their choice by any selfish or mercenary motives, forsaking their accustomed ways, and fondly embracing the most ridiculous vagaries as the only truth, can we explain such an occurrence in any other way, than by thinking of many other absurdities which have, for a time, been tolerated, admired, adopted, applauded, and then ridiculed, scoffed at, and despised.

Well has it been said by one of the great German poets, Goethe.—"For even when all ideas are awanting, a word or two will supply their place; with words you may fight most famously; with words you may build a system of philosophy; if man but hears words he will believe them." To give a specimen of the practical excellencies of homoiopathism, we cannot do better than allude to the course which has been pursued by the Russian Government towards it. A Saxon physician, M. Hermann, the great apostle of the system in Russia, was invested by the Grand Duke Michael with full powers to display, in a course of clinical experiments, its superiority over the common practice and theory of the day.

One of the wards of the Hôpital de Tuttschin, which contained a number of soldiers affected with fever and dysentery, was alloted to his special management, during a space of two months.

The following table exhibits the results.

| | Patients. | Cured. | Died. | Rem. |
|---|---|---|---|---|
| Common method, | 457.. | 364.. | —.. | 93 |
| Homoiopathic do. | 128.. | 65.. | 5.. | 58 |

It seems that the Grand Duke could use his eyes; he was satisfied, and withdrew his commission. However, some time after this the Ministers of the Russian Government summoned M. H. to Petersburg, gave him authority to select his own hospital, and to make any arrangements he thought fit. The wards were fresh painted, and every hygienic precaution faithfully executed. Even the kitchen was placed entirely under his control and superintendance; and in order to prevent the possibility of any interference a sentinel was placed before the door, and none permitted to enter during the occasional absence of M. Hermann. His first request respecting the patients was a very moderate and modest one, viz. that none should be sent to his hospital who laboured under ulcers, syphilis, dropsy, phthisis, &c. and that he should have the selection of all his cases!! Even under these most fortunate circumstances, the results were most unfavourable to the new practice; the proportion of deaths to recoveries was much higher than in ordinary practice, and the duration of the treatment was always protracted and tedious. Here is a specimen of the reports. The case is pronounced one of peripneumonia.

*History.* Patient ill for seven days; severe frontal cephalalgia, pains in the eyes, tongue clean, but dry, thirst, bitter taste in the mouth, anorexia: bowels open, dry cough during the night, and this is accompanied with pains in one side, which are increased on pressure; pains felt in the arms, skin cool, pulse full and not frequent.

An infinitesimal dose of arnica ordered. Next day but little change; but after this the symptoms gradually subsided, and the patient was discharged cured on the 19th day after admission.

A well-marked example of peripneumonia indeed!! A slight catarrh with an equally slight disturbance of the stomach. We might adduce fifty specimens of equally correct diagnosis, and astonishing success.—*Ann. de Hecker.*

"Extremes," it is said, "often meet," and the present day can boast of extremes and opposites flourishing beside each other without meeting. We have a nostrum-monger in this metropolis who has made his fortune by persuading John Bull and Jane Cow to swallow three, four, and six dozen pills daily for weeks and months continuously!—*Rev.*

---

### RUPTURE OF THE VAGINA DURING DELIVERY.

A healthy woman, about 30 years of age, and who had borne one living child, was the subject of this case. The labour had continued about eleven hours and had been rather difficult and tedious in consequence of an œdematous swelling of the labia, and of the lower extremities. The head had already descended fairly into the cavity of the pelvis, and the pains had become more severe, when on a sudden they ceased entirely; the patient complained of a sharp pain in the right iliac region; vomiting and general coldness succeeded, and the patient died in the course of an hour.

The different parts of the fœtus could be distinctly felt through the abdominal parietes. No outward hæmorrhage occurred.

*Dissection.* A small quantity of blood was found in the abdomen; the uterus empty, and contracted upon itself, was inclined somewhat to the right side, and presented in all respects a normal appearance. The fœtus had escaped into the cavity of the abdomen, and its body was enveloped among the intestines, the knees being placed against the abdominal parietes, while the head remained still in the passage, and was, as it were, incarcerated there by the edges of the opening, firmly applied round the neck. The laceration had taken place in the upper part, or vault of the vagina, which was enormously stretched, the child being unusually large. The placenta had descended into the vagina, and was partially protruded through the rent into the abdomen.—*Siebold Journ. der Geburtshulfe.*

---

Sur la Pathogenie de l'Axe Cerebro-Spinal. Par Dr. Rey. Quarto, pp. 110.

This book seems to have been written by a young medical man, who is determined to be an author, whether he has much to communicate or not. It is altogether occupied with the histories of cases extracted from a hospital record, and these histories are garnished with a few bald remarks.

The author becomes soon quite exhausted upon the announced subject matter, viz. the diseases of the "axe cerebro-spinal," and treats us with the account of an encysted tumor of the neck, of an impalement of the sexual passages in a female, and of divers other topics. We shall select one or two of the most instructive cases.

*Case of Hemiplegia—enormous Encephalic Effusion of Blood.*

A man of colour, between 60 and 70 years of age, of a strong constitution, but excessively addicted to intoxication, was suddenly, after one of his debauches, stricken motionless and insensible; the right side was quite palsied—the left exhibited slight muscular contractions. The pulse was less distinct in the right than in the left wrist; the actions of the heart were irregular and convulsive. He died on the following day.

*Dissection.* The scalp was so turgid with blood, that a large quantity flowed out when it was divided. The dura mater had a deep red colour, and all the superficial vessels were highly congested. On attempting to take the brain out, the quantity of blood that was found effused was equal to, at least, "deux saignées." The surface of the cerebellum was deeply ecchymosed. On the left side, the fissure of Silvius was almost obliterated by the distention of all the hemisphere around; and, at this part, a sense of fluctuation was perceived, as of an abscess. When cut through, a huge cavity, as big as two fists, and filled with dense coagula, was laid open. The left lateral ventricle was scarcely recognizable, in consequence of its cavity being occupied with a mixture of blood and medullary detritus. The septum lucidum was torn through, and thus a large hole of communication existed between the two ventricles. The right ventricle and the laceration, which opened into it, formed a cavity of the size of a man's fist—it was filled with fluid blood.

In some remarks appended to the preceding history, the author expresses his opinion that bloodletting, in such cases, always accelerates the fatal issue. The great difficulty consists in distinguishing encephalic rupture and effusion, from mere encephalic congestion. In the former case, blood depletions ought certainly to be more sparingly and more cautiously employed.

The only other case we shall extract abridged is one of—

*Encysted Tumor, situated on the Forepart of the Throat.*

A woman, 49 years of age, presented a large tumor, of the size of two fists, situated on the right side of the larynx and trachea. It was of 4 years' standing; but its growth had been much more rapid within the last, than during the preceding three. The voice had become, in consequence, very weak and shrill, and the sleep was disturbed, and any efforts of swallowing rendered difficult, from the sense of suffocation thereby induced. The distress in breathing was sometimes so great, as to amount to orthopnœa. The tumor, when examined, was found to be firm and resisting, and communicated an uniform elasticity: it extended from the base of the jaw to the clavicle, seeming to pass behind this bone, and to surround its sternal extremity; the larynx and trachea were pushed to the left side, and the sterno-mastoid muscle elevated and stretched. It gave no pain, and the skin over it was not discoloured. The internal and external use of iodine were tried, but with no further effect, than that a sense of fluctuation was afterwards distinctly perceived.

Dr. Rey considered the case to be one of encysted tumor, and proposed to open it, and to endeavour to excite inflammation of its internal surface, with the view of inducing adhesion of its walls. He first punctured it with a trocar, and gave issue to about ten ounces of a greyish, pultaceous ichor. Two days afterwards he made an incision, an inch and a half in extent, and discharged as much as on the former occasion: he then introduced three dossils of lint into the sac, and kept the lips of the wound apart.

Considerable fever supervened; the discharge was unhealthy, and very fetid, and the patient at one time became delirious. These symptoms, however, soon disappeared, and her condition was much improved, the neck recovering greatly its wonted freedom, in consequence of the contraction of the tumor. The wound not being kept well open by the lint, Dr. R. introduced a piece of silver catheter. The case went on progressively to a complete cure, which was pronounced complete in about three months after the operation.

Dr. Rey mentions that M. Guerin, a distinguished surgeon of Bourdeaux, has for many years past been in the habit of treating successfully all cases of hydrocele, by leaving the trocar in the wound for a quarter or half an hour after the fluid has been discharged; sufficient inflammation is thus induced to excite the adhesive process, and thereby to effect a permanent cure.

---

### EXTENSIVE MESENTERIC DISEASE—GREAT HEAT OF THE WHOLE BODY THE CHIEF SYMPTOM.

Giovanni L. æt. 36, a countryman, was admitted into the hospital at Padua in February 1830. His only complaint was a burning heat over every part of his body; and this was so distressing, that, although the weather was exceedingly cold, he lay all night without any coverings. Two bleedings from the arm, and cooling purgatives, relieved him so much, that he was able to return home. In the month of May he returned, labouring under the same distress, and was again made well by a similar treatment. At this time, however, some symptoms of a hypochondriacal affection were first observed.

A fortnight after his second dismissal he came back, in consequence of a mild attack of continued fever, accompanied with that feeling of burning heat which had so much distressed him before. A variety of remedies was tried, but with few good effects, the fever continuing in spite of them, and the patient gradually losing strength and flesh. The pulse being firm and hard, he was bled from the arm, and twelve leeches were applied round the anus. Being considerably relieved by this treatment, he was induced to leave the hospital. He was not, however, long absent, and, on his return, his condition was decidedly worse. The pyrexial symptoms were much aggravated, but he no longer complained of the burning heat; his emaciation was much greater.

Repeated examinations of all the great cavities of the body were made by more than one experienced physician, for the purpose of ascertaining, if possible, the seat or exciting cause of the prolonged duration of fever; but no very satisfactory conclusions could be arrived at by any one. It was suspected, however, that the "fons et origo mali" was probably seated in the lower part of the abdomen.

A diarrhœa came on, and, as it could not be checked, the patient speedily sunk under its effects. He died about the middle of August.

*Autopsy.* When the abdominal cavity was laid open, and the small intestines, which were found contracted and quite empty, had been pushed up, an extraordinary mass of indurated disorganized glands presented itself to view. It was fully as large as two fists put together, and, when divided, exhibited a yellow colour, and a structure not unlike to that of genuine scirrhus. So general was the morbid change, that not one healthy gland was discovered in the whole course of the mesentery. It was very naturally a subject of great surprise, how such an enormous enlargement as existed at one part could have escaped detection during life, seeing that the abdomen had been repeatedly examined with great care. The only way in which we can explain this, is by supposing that the intestines were always interposed between the mass and the parietes of the abdomen.

*Remarks.* The preceding case is very interesting in several points of view; and, of these, not the least important is that of illustrating what extensive disorganization may be going on in certain viscera, and yet the symptoms, especially the local ones, may be very obscure and unsteady. The leading feature, in our patient, was the extreme heat and sense of burning which he felt in every part of his body.

It may, therefore, be worthy of the attention of physicians, whether this symptom is not more frequently attendant upon mesenteric disease than has been noticed hitherto. We have certainly observed it, more than once, in some of the abdominal affections of children.—*Annali Univers. di Medicina.*

---

### Incurable Neuralgia of the Ulnar Nerve.

Professor Viviani, not long after his recovery from an attack of rheumatic sciatica, began to experience slight pricking and formication, preceded by a feeling of an aura in the left forearm, along the line of the ulna, the ulnar side of the carpus, and in the little and ring fingers. These sensations, at first trifling and transitory, became more severe, and fixed in the palmar superficies of the wrist, on the side of the pisiform bone, and extending thence along the palm to the two fingers, but never retrograding. The paroxysms of pains increased in violence—sometimes short, but dreadfully agonizing, each throb shooting along with the rapidity of lightning; at other times, they were more protracted. The pulse at the part was never affected, and the general health of the Professor was perfectly good. During the space of three years, every remedy that could be devised was tried, but in vain. He then consulted the celebrated Scarpa, who gave it as his opinion, that the nerve was probably not diseased higher up than the seat of the pain, as he found that firm compression on the carpus, during a paroxysm, very considerably mitigated the pain. He, therefore, recommended the division of the nerve in the forearm.

An incision was made, beginning about an inch above the pisiform bone, and carried upwards, along the side of the tendon of the ulnaris internus; and, on dissecting between this tendon and that of the palmaris longus, the ulnar artery and its accompanying nerve were easily exposed. About half an inch of the nerve was excised; the little and ring fingers immediately lost all feeling and mobility.

The hopes of the patient and surgeon were soon blighted. During the night after the operation, a paroxysm, quite as severe as any preceding one, was experienced; the pain seemed to commence at the upper angle of the wound, and darted to the extremities of the two fingers. The condition of the patient was, therefore, not at all bettered by the operation; indeed, the fits of suffering became more lengthened and excruciating; on one occasion, the pain lasted unceasingly for 36 hours. The two fingers were all this time quite palsied, and generally half bent upon the palm of the hand.

Four years after this date, Professor Viviani wrote to Scarpa, acquainting him that, since the operation had been performed (1827), his sufferings had become more and more intense, and that they had defied every attempt, even to relieve them for a time.

### General Remarks on Neuralgia. By Scarpa.

He very justly remarks that there are two sorts of neuralgia, very different from each other in almost every respect, and yet which are daily and hourly confounded with each other.

The one, "neuralgia anomala," of Chaussier, proceeds or arises from a local and circumscribed cause, which may in most cases be removed by the hand of the surgeon. The other, "neuralgia essentialis, vel legitima," appears to depend upon some cause, perhaps constitutional, which we cannot appreciate, or discover, and very generally defies all medical and surgical treatment. The latter species is usually seated in some of the most superficial nerves, returns at irregular intervals, induces little or no change in the appearance of the part affected, either during life or when examined after death, and may therefore be supposed to have a constitutional or systemic origin, and not merely a local and circumscribed one.

Scarpa combats the idea that every case of neuralgia is dependent upon an inflammatory state of some portion of the nervous system. This is quite a gratuitous assumption. He thinks it more probable that its etiology is somewhat analogous to that of epilepsy, (although he is too cautious to conjecture what the essence of either disease may be) and proposes to call it "epilepsia sympathica partialis."

How are we to explain the recurrence of the neuralgic agony in a part which has been palsied by the section of its nerves? We do not pretend to solve the problem, but shall merely hint, that it appears to be a phenomenon very much akin to that of the recurrence of pain in the situation of a limb which has been amputated.

In conclusion, Scarpa observes that we have reason to anticipate more advantage from constitutional, than from local treatment, in cases of legitimate neuralgia; for sometimes nature alone will, after years of suffering, bring about a mitigation, or even an entire dissipation of the pain.—*Ibid.*

---

### ADHESION OF A SEPARATED FINGER—PARTIAL GANGRENE—RECOVERY.

A medical student accidentally divided the last phalanx of his left fore-finger so completely across, that the divided piece fell on the ground. It was immediately replaced, and secured by adhesive straps and a bandage. On the fifth day afterwards the dressings were removed, and it was then found that gangrene had taken place in the truncated phalanx; but most fortunately this did not extend beneath the surface, for when the mortified cuticle was removed, the subjacent textures were of a red colour and seemed quite healthy; adhesion, although somewhat imperfect, had taken place: in other three days it was complete.

The sensibility of the apex of the finger was for a length of time very feeble; but this was gradually restored, nearly to its normal standard.

The preceding history furnishes an apt corroboration of the accounts which have been left us by Tagliacozzi and others; although Richerand and some other moderns have attempted to ridicule them as unworthy of credit.

Dr. Angelo della Cella, the reporter of this case, with much good humour invites M. Richerand and his followers to cross the Alps and pay him a visit on the banks of the Entella, when they will be able to see with their own eyes, and then try to believe.—*Ibid.*

---

### CASE OF PARAPLEGIA—SUPPRESSION OF THE URINARY AND ANAL EVACUATIONS DURING ELEVEN YEARS.

When Dr. Montesanto first saw this patient in April, 1831, he had been paraplectic for upwards of eleven years, and was suffering at the time from the sequelæ of a severe attack of pneumonia; so that it was not expected that he could long survive. The thoracic symptoms however gradually disappeared, and he was then in the same paralytic condition in which he had remained since the year 1820. His appetite was vigorous; but the food seemed to meet with some obstruction, probably at the pylorus, for it was regularly rejected by vomiting in about three hours after it had been swallowed. It is stated that at a former part of his illness a stercoraceous vomiting, which had previously recurred at intervals of from forty to fifty days, had ceased altogether for more than two years. No secretion of urine, nor evacuation per anum had taken place since the commencement of the disease, in 1820; neither during all these years had there been any traces of activity in the generative organs. Strange that with such a defect and morbid state of the alimentary and urinary organs, the general health of the patient should have continued moderately good. Towards the close of the year 1831 he had a threatening of the return of his thoracic complaints; but they were speedily removed by appropriate treatment. Occasionally too, when he eat any food which disagreed with his stomach, or took it at improper times, he was seized with alarming symptoms of cramp and ineffectual efforts at vomiting. One of these attacks which had been brought on by a repast of fried sardinias, nearly proved fatal in the Spring of 1832; for fifteen days his life was despaired of; fortunately then a spontaneous vomiting occurred by which he rejected four large masses of solid stercoraceous substance, which seemed to have been impacted in the intestines. Nature having thus relieved herself of an immense accumulation of fæcal matter, which had been gradually collecting for a space of nearly three years, the health of the patient was speedily restored to its former condition. It was necessary every now and then to take away a small quantity of blood by venesection, to counteract in some degree the stimulating and plethoric effects of the ardent spirits which the patient was in the habit of drinking.

In the Summer of the following year Dr. Montesanto makes the report, "that his patient's health for the last twelvemonth has been on the whole exceedingly good; and that there has been no return of the stercoraceous vomiting during that period."

The paralytic state of the lower half of the body remained unchanged; all sensibility quite gone, but the limbs not wasted, and though motionless at will, were supple and flexible.

The authentic particulars now related have attracted the attention of many of the most distinguished physiologists and surgeons in Europe; the case altogether is one of the most wonderful on record—the mode of existence in this man being allied to the normal condition of life in some of the lower classes of animals.—*Annali Univ. di Medic., Agosto*, 1833.

---

Case of apparent death [life?] which lasted for three weeks.

A young man who had recently been cured of a tertian fever, was admitted into the hospital at Paderborn, under the care of Dr. Schmid, for symptoms indicating tubercular phthisis. He gradually became exceedingly emaciated, and at length died.

After all traces of breathing had ceased, a few irregular beats of the pulse were felt, and the eyes opened of themselves. Some small eschars artificially produced exhibited signs of suppuration on the second, third and fourth days. On the fifth, one hand was found to have been turned round; and on the sixth and ninth days a partial perspiration bedewed the skin. After this period several pemphigus-like bullæ made their appearance. The limbs remained quite pliant; the lips preserved their red colour until the eighteenth day, and the expression of the features even at this date was that rather of a living than of a dead person. At the end of the third week there was no offensive smell nor any other sign of putrefaction. —*Journ. der Pract. Heilk.*

---

Preservation of Leeches.

M. Bertrand recommends that leeches after they become detached from a part should be gently passed between the thumb and fingers, (or, as it is vulgarly called, stripped) so as to make them disgorge most of the blood which they have sucked, and then be put into water moderately sweetened.

In this method leeches, we are told by M. B. may not only be kept for many years, but will retain their activity, and be fit for reapplying every third or fourth day.

Dr. Scheel has more recently recommended the following treatment as still more effectual.

The leeches should first be put upon a warmish plate, and be well besprinled with carbonate of soda; when they have disgorged most of their blood, they should be washed several times with tepid water, then put again upon a plate and some sugar sprinkled upon them; and lastly be washed with cold water and put into a large vessel full of it, gently sweetened.

The sugared water must be changed once a day, or oftener if it becomes discoloured. Those leeches which die or even become wrinkled and faded, should always be cast away.

Dr. S. has succeeded in preserving leeches thus for a great length of time, in good health, and in a condition ready for use every six or seven days.

When there is a very great dearth of leeches, the following expedient may be had recourse to. With very sharp scissors we may cut them right across, somewhat nearer the tail than the head, and then apply them as usual. As the animal sucks, the blood trickles from the divided end. As a matter of course the attendants must be on their guard to prevent an excessive discharge by detaching them at a proper time. They are then to be gently squeezed and put into fresh water. If the divided extremity should become agglutinated together and closed, we must employ a rather firmer pressure from the mouth downwards, so as to cause the blood to force an exit. Leeches which have been mutilated in the manner above mentioned, have been used several times every day for many weeks.—*Allgemeine Medic. Zeitung.*

---

### INJECTIONS OF COLD WATER INTO THE UMBILICAL CORD TO PROMOTE THE SEPARATION OF THE PLACENTA.

Professor Hohl states that this method, first recommended by M. Mojon, will often succeed perfectly if it be used sufficiently early, and provided the vein does not contain too much blood; for sometimes cases occur in which it is not possible to dislodge the blood which the vein contains. One injection will frequently suffice. If we listen with the stethoscope over the region of the womb, where the placenta is attached, while a quantity of water is injected into the cord, a noise or bruit coming as if from a distance is heard; this noise is quite distinct from that of any pulsation. In favourable cases the sound becomes louder and stronger, and in addition to it, other bruits of a more sibilant or whistling character become audible. Professor H. states, that he never could hear any of these last described sounds in cases in which the placenta remained obstinately attached; they would therefore seem to be connected with the contractions of the uterus. These contractions are necessarily very imperfect, when the placenta remains fixed; and hence perhaps the absence of the sounds in these cases. Professor H. recommends that the injection of the cord be used even when it does not succeed of itself, and when therefore it is necessary to remove the placenta by manual assistance; it may be a serviceable adjunct.

The most frequent cause of failure with the injections alone, is spasmodic contraction of the uterus.—*Ibid.*

---

### REMARKS UPON AN ORGANIC DISEASE OF THE LUNGS, PRODUCED BY THE IMPERFECT RESPIRATION OF NEW-BORN INFANTS.

If the respiration of a new-born infant be, from whatever cause, impeded, or rendered incomplete, the circulation of the blood, must, it will be allowed by all, necessarily be disturbed. This disturbance of the course of the sanguineous fluid is the cause, according to Dr. Joerg, of an agglutination, or "coalition," of the pulmonary cells, over a greater or smaller extent of the lungs proportionate to the amount of the morbific cause.

This diseased state is rarely the result of inflammation, or of its consequences; but solely of the non-permeation of a portion of the pulmonary tissue by the air; the cells become more and more contracted, and finally are quite obliterated. The portions of the lungs so affected sink in water, in the same manner as hepatised or solidified lung from inflammation. One of the most common causes of this diseased state is undue quickness of the delivery. It is a fact well known to accoucheurs, that when a child is very rapidly ushered into the world, the first act of breathing is often delayed longer than is common, and the child appears to suffer; the circulation of the blood is consequently disturbed at the same time, and the foundation of a morbid state of the lungs is laid.

Another cause is pressure on the cerebellum, either from the immoderate contraction of the uterus, or from the forceps, when the blades are too tightly compressed: a state approaching to asphyxia is thus induced, and the functions of respiration and circulation obstructed. The child is observed to be weak, and moves the limbs feebly; the limbs hang as in a palsied patient; the voice is thin and plaintive; the eyes are half open, and the movements of the chest are indistinct, and either hurried or unusually slow. By the use of the warm bath and other means the child may be considerably revived, but the breathing remains oppressed, and each act is short, such as we observe in cases of asthma and of hydrothorax.

This description applies to those infants who are too quickly expelled: those on the other hand who are subjected to immoderate pressure usually exhibit a tumefaction of the head, a livid state of the skin, and most of the appearances of an apoplectic patient. Sometimes the breathing is not established for 15 or 20 minutes; and then it may be only imperfectly, and the child remains exceedingly weak and enfeebled. Convulsions are not unfrequent under such circumstances. When it cries the voice is puny and moaning, and the movements of the chest are at one time scarcely perceptible, and at another heaving and irregular. The livid colour of the skin becomes less, but either does not vanish altogether, or is exchanged for an unhealthy pallidity, and there is often a cold perspiration on the surface.

The mouth and eyes are usually half open. When the disease is likely to prove fatal, the skin, from being pale, becomes suddenly blue; the child is so weak that it cannot suck, or even swallow; the voice is scarcely perceptible, the pupils are motionless, the head is drawn backwards, and a slight rattling is heard in the throat. However alarming the symptoms may seem to be, they never prove fatal on the first attack; but very generally may be mitigated by appropriate treatment, the strength becoming somewhat restored, and the convulsive or spasmodic state much lessened. Perhaps the subsequent attacks may be gradually milder, and the child almost perfectly recover; but if, on the other hand, the weakness should increase, we can have small hopes of saving the little patient. Even when it does recover, the continuance of life is always precarious; for not unfrequently a bronchitic affection, such as the "catarrhus suffocativus," supervenes and rapidly proves fatal. The partially obstructed state of the pulmonary tissue adds greatly to the danger of such attacks. The child dies suffocated, or with all the symptoms of pressure on the brain.

Dr. Joerg is of opinion that many cases of permanent morbus cæruleus are attributable to the impediment offered to the freedom of the circulation by the obstructed state of the lungs.

We may enumerate this very generally mortal affection of the heart as a fifth mode, in which the disease of the lungs usually proves fatal; the other four being apoplexy, suffocative catarrh, bronchitis, and lastly atrophy.

Our prognosis must be in most cases unfavourable; but it will, as a matter of course, be regulated by the extent of the injury which the respiratory function appears to have sustained. Of this we may form a tolerably accurate notion by attending to the breathing of the child, and observing whether it is ever full and deep; and also by the loudness of the voice in crying.

In endeavouring to resuscitate the child at first, great caution should be used, that the system be not over-stimulated by the means employed, for a corresponding exhaustion will inevitably succeed to such a state; and besides, if there be any encephalic congestion, the evil will be aggravated. The tartar-emetic, judiciously given, may very possibly be useful, especially when the head is free from any injury, and the affection of the lungs is, therefore, more idiopathic. The warm bath, too, will be found a good adjunct; and in cases where the infant is so weak that it cannot suck, we may immerse it in a bath of tepid milk. Artificial respiration will be necessary in some cases.—*Allgemeine Medic. Zeitung.*

---

### TREATMENT OF PORRIGO.

Dr. Cazenave observes that, unless our patients be scrofulous, or otherwise cachectic, the constitutional treatment resolves itself into the mere use of an occasional purgative and of a tepid bath. An indispensable part of the local management is cleanliness. The hair must be kept closely shaved, and the scalp frequently washed with emollient, alkaline, and sulphureous lotions, which have the good effect of separating the crusts, and cleansing all nastiness away. Dr. Biett highly praises the following formulæ.

℞. Potassæ sulphuret. ʒij.

Saponis alb. ʒiij.

Aquæ calcis, ℥viij.

Spirit. vini rectif. ʒij. Misce fiat lotio.

And—

℞. Lythargyri, ℥ij.

Aluminis usti, ℥jss.

Calomelanos, ℥jss.

Adipis suis, ℔ij.

Terebinth. venet. ℥vj. Misce.

A still more effectual remedy is the ioduret of sulphur which may be used thus—

℞. Ioduret. sulphur. ℈j.—ʒss.

Adipis suis, ℥j. Misce.

Of this ointment, a drachm may be rubbed in each time.

The use of caustic, as of nitrate of silver, is to be recommended only when the disease is confined to one spot, or two.—*Bullet. Gener. de Therap.*

---

### HÆMORRHAGIC TENDENCY IN A FAMILY.

The mother was of a delicate constitution, and liable to profuse menorrhagia. She was delivered of a boy in March, 1829. It seemed tolerably healthy, although fair, or almost white.

It suffered successively from jaundice, aphthæ, convulsions, and a diarrhœa, which proved fatal on the ninth day after birth. The navel-string was constantly moist with a bloody serum, which kept oozing from its divided extremity, and resisted every styptic. When the tied portion fell off, pure blood trickled from the umbilicus, which was thus always covered with a coagulum. The scrotum, in this infant, had a dark purplish colour. Three former children of this woman died on the 8th, 12th, and the 14th days after birth, in consequence of bleeding from the navel.

The other three children of the family, although delicate, did not exhibit any hemorrhagic tendency.—*Journ. der Pract. Heilk.*

---

### CASES ILLUSTRATING THE TERMINATIONS OF OVARITIS PUERPERALIS.

The epithet puerperal, applied to this affection, is not to be considered as indicating that it occurs only after delivery. In the 4th vol. of the "Clinique des Hôpitaux," is a report of the dissection of a woman (who never had been pregnant), in whom the right ovary was found inflamed and much enlarged, from a purulent deposit, of a most fetid character. In another case, occurring under similar circumstances, the pus made its escape by the rectum, and the patient recovered.

In many of its features, ovaritis bears a strong resemblance to the abscess of the iliac fossa, the history of which has been so ably illustrated by Dupuytren, Dance, and others. At present, we shall confine our observations to the mere furnishing of cases, descriptive of the different modes in which ovaritis may terminate.

1. *By Resolution.* A woman, 33 years of age, was admitted into the Hôtel Dieu on the 15th day after delivery. The labour had been painful, and the child extracted by turning. The symptoms were œdema of the abdominal parietes and of the inferior extremities, suppression of the lochia, a swelling in the left iliac region, painful on touch; strangury; whitish, creamy deposit in the urine, and sense of weight in the vagina. By active local bleeding, and appropriate constitutional treatment, this woman speedily recovered.

2. *By Suppuration.* This is a very frequent termination. The pus makes its escape either, *a*, by the rectum, as in the following case.

A young woman presented a general emaciation—slight effusion into the cavity of the abdomen, enlargement of the liver, a swelling, as large as a hen's egg, in the left iliac region, painful on pressure—amenorrhœa—urine containing a whitish substance, which appeared like pus; well-formed pus mixed with the stools: body and neck of the uterus healthy to the touch, by which it was discovered that the tumor in the groin was connected with the womb and bladder. The patient was generally feverish, and more so towards evening. Numerous leeches were applied to the swelling, and hot fomentations afterwards. The progress of the case is not known, as she left the hospital unexpectedly. It ought, however, to be stated, that she had been delivered of a seven-month child three years before her admission, after a severe, but rapid labour. Three months after this date, she began to experience pains in the hypogastrium and groins, and these had continued with more or less severity ever since. In such cases of purulent diarrhœa, the pus may make its way either into the cæcum, the arches of the colon, or into the rectum. In the following case, it seems to have escaped into the left arch of the colon.

A woman was seized, on the second day after delivery, with all the symptoms of peritonitis; on recovering from which, she had an attack of phlegmasia dolens. While under treatment for this, a painful tumor made its appearance in the left groin. This attack of ovaritis was no sooner over, than she was again seized with peritonitis, in consequence of imprudently walking on a cold stone floor. On the 22d of March (seven weeks after her admission into the hospital), the left limb was still œdematous; and on this day was first observed purulent matter, mixed with the alvine evacuations. The great relief which the patient almost instantaneously experienced in the inguinal swelling, on the occurrence of this purulent discharge, could leave no doubt but that it proceeded from the ovary, which had very probably become adherent to the sigmoid flexure of the colon. During a space of two months the discharge ceased, and returned several times; and even when the patient left the hospital, on the 23d of June following, there still remained a degree of engorgement in the left groin, and slight œdema of the limb.

*b.* The pus may find an exit by the bladder or vagina. MM. Husson and Dance found that this had taken place, in a young girl who died of the disease.

*c.* It may follow the course of the round ligament, and escape at the inguinal or crural apertures. Dupuytren has seen numerous cases of such a termination. Under these circumstances, the tumor may be mistaken for an aneurism, as it frequently pulsates, from being in close proximity to the iliac artery. In opening abscesses at this point, it is necessary to use considerable caution, as instances have been known where the artery has been inadvertently wounded.

*d.* It may pass into the abdominal cavity, and either become encysted, or induce fatal peritonitis : and, *e*, lastly, it may be discharged at some point of the hypogastric or iliac regions (besides the inguinal aperture), in consequence of the ovary becoming adherent to the abdominal parietes, and the matter gradually working its way out. This termination is illustrated by the following case.

A woman, twenty-four years of age, was delivered of her sixth child on the 17th Nov. The labour was rather painful and difficult. Imprudent exposure to cold was quickly succeeded by an attack of fever, by suppression of the lochia, and a tumefaction of the right groin. When received into the hospital, the tumor was of the size of an egg, and the limb was œdematous.

In spite of repeated leechings, &c. the suppurative process commenced, and, by the end of January, several fistulous openings through the abdominal walls had taken place; and from these a copious discharge of pus flowed out. The patient gradually regained her health, and left the hospital, quite cured, a few weeks afterwards.

In the 4th vol. of the Bibliothêque Médicale is narrated the case of a lady, in whom two iliac abscesses, supervening upon an attack of entero-peritonitis, opened, the one into the sigmoid flexure of the colon, the other into the cæcum—and this last also projected outwardly. An incision was unfortunately made into it, and a stercoral fistula was the consequence.

3. *By Ramollissement.* The ovary becomes tumefied, infiltrated with a sero-purulent fluid, and either friable and easily lacerated, or extremely soft and yielding in texture. Dr. Montault saw an example of this degeneration in a young girl, who died of puerperal peritonitis. The labour had been quite natural and easy, but she had suffered much from mental anxiety, and had been exposed to cold, when she was brought to the hospital after delivery.

4. *By Enlargement and Induration.* A young woman was seized with metro-peritonitis, five days after her discharge from the Maternité, where she had been safely delivered. She died on the sixth day of the disease, having, on the day or two preceding her dissolution, exhibited all the symptoms of ataxic fever (from the absorption of purulent matter into the system.) On dissection, a small quantity of pus was found infiltrated into the superior and lateral portions of the uterus. The right ovary was more enlarged than the left, hardened in texture, and of a yellowish colour; firm pressure forced out only a few drops of pus. This state of induration will often continue for a long period without affecting the general health; although it must be confessed that, not unfrequently, the patient is annoyed with colicky pains, proceeding from the site of the ovary, with dysmenorrhœa and other troublesome symptoms.

When these are exceedingly obstinate, and progressively become more distressing, we may suspect that the enlarged and hardened viscus is degenerating into a scirrhous, lardaceous, osseous, melanotic, or hydatidic condition.—*Journal Hebdomadaire.*

---

### Case of Cyanosis, occurring on the Ninth Day after Birth—Cure.

The infant, when born, appeared quite healthy and strong, and indicated by its lusty cries a vigorous vitality. It exhibited not the slightest trace of any cutaneous discoloration, nor of any impediment in the breathing.

The mother being unable to suckle her child, it was reared with spoon food; but in the course of a few days, symptoms of disordered stomach and bowels, announced by restlessness, fever, green stools, and griping, set in. These were for a short time relieved by carminative medicines, by rubbing the abdomen with an oily balsamic embrocation, and by camomile enemata. On the ninth day, the infant became much worse, and to the already-mentioned disorders were added convulsions, and an unexpected invasion of "morbus cæruleus" over the whole surface of the body and limbs. Every part, including even the tongue and nails, were stained of a deep blue colour. The tender patient seemed to suffer much distress, as it kept constantly moaning most piteously, and drawing its limbs convulsively together. The temperature of the surface was somewhat lower than in health—the pulse was small and contracted, but not quickened—the breathing was short, feeble and irregular, deep at one moment, and hurried the next.

Whatever opinion might be formed of the proximate cause of the purple colour, it could not be overlooked that its invasion had been preceded and accompanied by a convulsive state of the system, arising from severe colic: moreover, it could not reasonably be attributed to any transitory retardation of, or obstruction to, the course of the blood, else it must have speedily passed away; and we were, therefore, obliged to suspect that, in consequence of the pulmonary circulation being disturbed, and thus occasioning an excessive accumulation of blood in the right cavities of the heart, during the recurrence of the repeated spasms, either the foramen ovale or the ductus arteriosus had been forced open, and allowed the admixture of the venous with the arterial blood. The prognosis was, therefore, most unfavorable; and the indications of treatment were, to correct the disordered state of the bowels, and to allay the general distress.

For this purpose, a tea-spoonful of the following mixture was ordered to be given very frequently:

℞. Magnes. carbon. ʒj.

Aquæ fœniculi, ℥j.

Mucilagin. Arab. ℥ss.

Moschi Orientalis, gr. vj.

Syrupi rhæi, ℥ss. M.

An occasional warm bath and assafœtida enemas were also prescribed. Little good was derived from these remedies, and, as the child evidently began to be weaker and more exhausted, a nervous powder, containing one grain of musk, was given every hour or two. This treatment was continued for two days (so that 26 grains of the musk were taken in this period), with manifest advantage; the heat of the body became greater, the circulation and breathing less disturbed, and the child more lively and comfortable—the blue colour, however, was unabated.

But, even in this symptom, a favorable change soon began to be perceived. The musk was persevered in, one grain being given every three or four hours, and a tea-spoonful of the magnesia and rhubarb carminative occasionally. An aromatic bath was also used twice a day.

The case went on progressively to a complete cure; the blueness of the skin gradually subsided, and altogether disappeared about the 12th day from its invasion.—*Journ. der Pract. Heilk.*

---

### On the External Use of Croton Oil.

This valuable drug was first made known to the profession by Dr. Connwell, in 1820, and subsequently its therapeutic effects were investigated by MM. Recamier, Bally, and Majendie; their researches were, however limited to its internal exhibition, and it was not until 1831 and 1832 that its great value, as a counter-irritant to the skin, was clearly proved by Professor Andral.

*External Use.* With one or two fingers, or, if we choose, with a dossil of lint, wetted with the oil, we continue rubbing the skin for the space of about 10 minutes. This operation should never be entrusted to the patient himself—in two cases at the Hôpital de la Pitié, we observed violent ophthalmia and inflammation of the penis and scrotum induced, no doubt in consequence of the mere inadvertently carrying their fingers to their eyes and genital organs.

The eruption, which is brought out by the external use of the croton oil, may be divided into five stages: 1, Rubefaction of the skin—2, Formation of vesicles—3, Conversion of the vesicles into pustules—4, Desiccation of the pustules—5, Desquamation and falling off of the crusts.

These different periods or stages are not uniformly to be observed; they are most conspicuous when the friction has been made with ten or twelve drops of the oil, on a part of the skin where there is much subjacent cellular tissue. The patient at first experiences a tingling warmth, which is quickly followed by a considerable redness, extending an inch or so beyond the sphere of the rubbing. These appearances are generally noticed within 7 or 8 hours, sometimes in 1 or 2, at other times not for 10 or 12 hours; the differences of time required depending, no doubt, on the delicacy of the skin. In from 14 to 26 hours, myriads of small, close-set vesicles make their appearance upon the inflamed skin. Occasionally, a few of the vesicles become greatly magnified, forming true phlyctenæ, filled with a turbid lymph, which speedily change into a purulent matter. In 12 out of 31 cases, reported by our author, the vesicles passed to desquamation without undergoing the suppurative process.

The usual period at which the serum becomes puriform, is from 36 to 54 hours after the application of the oil. In one or two days subsequently, the pus begins to exude, and forms greyish crusts over the pustules, and the desquamation is generally over by the 8th or 9th day. If the croton oil is rubbed upon any part which has been recently vesicated, the eruption is, as we might expect, more speedy and abundant.

In six cases, it was tried whether the rubbing in of the croton oil, mixed with an equal quantity, or with rather more, of that of almond oil, over the arch of the colon, would produce any purgative effects;—an eruption, which reached the second stage, was brought out, but the action of the bowels was not affected. Similar results were obtained when the pure oil, to the amount of 20 drops, was rubbed round the umbilicus. Dr. Rayer states that he has repeatedly induced free action of the bowels, by putting two or three drops of the oil upon a surface, denuded of its epidermis by a blister. We have not repeated this experiment.

*Therapeutic Effects.* The diseases in which the external use of this remedy has been employed with most advantage, are chronic rheumatism, arthritic pains, pleurodynia, paralysis, stomatite, laryngitis, and chronic gastritis.

CASE 1.—*Sciatica.* A man, aged 48, was admitted into the Hôpital de la Pitié on the 6th Dec. 1831. For five months preceding, he had suffered severely from pain, beginning in his right hip, and extending down the back of the limb, along the course of the sciatic nerve to the outside of the leg. For two months and a half he was obliged to keep the house, and, upon then attempting to resume his work, the pain returned with all its former intensity. He attributed his complaints to exposure to wet and cold. The only treatment which had been followed before his admission was blistering the limb; but he had derived no benefit. When examined in the hospital, the pain was found to be increased by pressure, and by the heat of the bed; he complained of headache, but in other respects his general health was not amiss. Eight drops of croton oil were ordered to be rubbed in over the origin of the sciatic nerve. This produced considerable itching and redness, but no vesicles; and, the pain being not relieved, 18 drops of the oil were rubbed along the whole trajet of the affected nerve. Next morning, the outer side of the leg was much reddened, and vesicles had formed over the trochanters. On the 11th, ten drops more were rubbed in between the trochanters.—12th, The eruption considerable—some large papulæ had appeared over the fibula. The neuralgic pain almost gone—only the heat and itchiness of the eruption are troublesome. He left the hospital in a few days, quite well.

CASE 2.—*Sciatica.* A man, aged 50, entered the La Pitié Hospital in Dec. 1831, suffering from sciatica of six weeks' standing. Twelve drops of croton oil were well rubbed in between the trochanters, along the outside of the thigh, to the lower third of the leg; a copious eruption was induced, and already, upon the second day, the patient felt relieved. In six days more he was considered cured, and left the hospital, quite delighted with the rapidity of his cure.

He had experienced two severe attacks before—once in 1812, when he was treated in the Hôtel Dieu by M. Recamier, with the essence of turpentine—at that time he was six weeks in the hospital; and again, two years ago, after exposure to wet and fatigue. he was admitted into the Hôpital de la Charité, under the care of M. Fouquier, who employed blisters and friction, with anodyne balsam. He was cured then in three weeks.

Case 3.—*Sciatica.* A stout, plethoric man, 45 years of age, had, for about a month, felt general indisposition, frequently-returning shiverings, and neuralgic pain of the left extremity. He was taken into the La Charité Hospital, and there treated by M. Rayer with repeated venesection, the application of 50 leeches to the hip, and 40 more to the back of the thigh. The essence of turpentine was administered inwardly in frequent doses; and besides all this, the vapour-bath was used fourteen times. This treatment was continued for three weeks, and, as little benefit had been obtained, the patient left the hospital, and a few days subsequently entered the La Pitié. At that time, the pain extended from the ischium down to the ankle-joint, and it was increased by walking, and by the heat of the bed : the lower part of the leg was annoyed with a feeling of formication. The digestive organs were in good order. Fifteen drops of croton oil were rubbed in over the origin of the sciatic nerve. On the following day, 20 drops more were rubbed over the tract of the affected nerve; a vesicular eruption made its appearance, and the neuralgic pain was already diminished. On the 29th (3d day), twenty drops were again ordered. 30th. The eruption is very abundant—the vesicles have changed into large pustules. The patient complains only of the itching; the pain is gone. He remained a few days longer in the hospital, until the crusts separated; and, on the 12th day after his admission, he was discharged cured.

A case of chronic rheumatism of the shoulder-joint, supervening on typhus fever, is given, in which general and local bleeding, blisters, &c. had been fruitlessly used for the space of six weeks. The friction with a few drops of croton oil was employed twice; and on the third day the patient could move his arm—although not entirely cured, he was very much relieved, when he left the hospital.

Case 4.—*Anæsthesia, or Paralysis of Feeling.* Pierre Dumas was admitted into the Hôpital de la Pitié on the 9th Nov. 1831. Seven months before he had an attack of erysipelas of the face, and the inflammation had extended down the left side of the neck. Three weeks after his recovery from this illness, he was suddenly seized with dimness of sight and stunning noises in his ear; these symptoms were not constant, but came and went, returning every 2d or 3d day.

This state of things lasted for about two months, during which nothing had been done in the way of medical treatment; and then there supervened a general numbness of the whole left side of the face, and the sight of the left eye became affected at the same time—the left nostril lost the sense of smell, and the left side of the mouth its sense of taste. When he was shaving, he felt as if some foreign body was placed upon his cheek; and, in chewing, the food seemed like earth in his mouth. He complained of a very severe frontal cephalalgia—the tongue was loaded, the abdomen soft, the bowels rather relaxed, pulse 90, breathing not affected. He was ordered to be largely bled from the arm—to use a mustard-bath to his feet at night, and to be put on a light emollient diet.

10th. No relief; half a drop of croton oil in two pills at bed time.

11th. Twenty liquid stools from the pills; headache much better; paralysis not affected. Eight drops of the oil to be rubbed upon the left cheek and side of the neck.

On the following day the skin was well reddened, and a large crop of confluent vesicles had made their appearance; to our great surprise he had already recovered his sight, taste, smell and feeling. The fifth period of the eruption, or that of desquamation, was not over until the thirteenth day. He did not leave the hospital till the 12th December, having had a threatening of a relapse of the numbness in a slight degree; but this was checked by a bleeding from the foot.

Case 5. *Angina Laryngea—Aphonia.*—An itinerant singer of the streets of Paris, presented, upon his admission, the following symptoms; a frequent dry and harsh cough: pain over the larynx, increased by swallowing; breathing sibilant; voice almost entirely gone, so that he could not make himself understood. Upon examining his throat, the velum and its pillars were observed red and swollen. Venesection, a sinapised foot-bath and emollient drinks ordered. The following day he was much better; the general symptoms were relieved; but the aphonia was as complete as before. Ten drops of croton oil to be rubbed on the front of the neck. In 24 hours there was a copious confluent eruption; the voice was regained, and the deglutition more easy. He left the hospital three days afterwards quite cured.

Case 6. *Diptherite, or Stomatitis pseudo-membranacea.*

An old soldier, upwards of 60 years of age, exhibited a specimen of this disease to our notice in the Hôpital de la Pitié.

It had already existed for eight days, and had made considerable progress; the inside of the mouth and the surface of the tongue being covered every here and there with small oblong crusts, or laminæ of a greyish-white colour, set upon red, inflamed, and swollen bases; the submaxillary glands were painful and enlarged; the breath excessively fœtid, the lips swelled and of a purple hue; and the deglutition very difficult. Sixty leeches had been applied at two different times behind the jaws; and poultices and a multitude of gargles had been used without much good for the poor patient, who could scarcely articulate a word. Eight drops of croton oil were ordered to be rubbed in upon the sides of the neck. On the morrow a copious eruption of vesicles had appeared, and considerable relief was already experienced. From this period the disease appeared to have undergone a favourable change, and in fifteen days more, under the use of acid gargles and of poultices, it was altogether removed.

---

### Causes of the Shortening of the Limb in Morbus Coxarius.

M. Dzondi, in a remarkably perspicuous essay on this disease, observes, that the shortening of the limb which takes place in the third stage of hip-joint disease, is not attributable solely and on all occasions to the actual dislocation of the head of the bone from its cotyloid cavity. It may be produced, to a certain extent, by other causes. Among these he enumerates—

1. Sympathetic irritation of the cervix femoris, occasioning an alteration in its direction; so that the angle forming between it and the shaft of the bone becomes more acute.
2. The extenuation or destruction of the articular cartilages and of the synovial membranes.
3. The irregularity and unevenness of surface which the head of the bone and the acetabulum acquire, and which prevent their accurate contact.
4. The os femoris itself undergoing a certain change, and becoming somewhat shorter.
5. The perforation of the fundus of the acetabulum, and the protrusion of the ball of the former through it.

In an earlier part of the essay M. Dzondi has most accurately described the symptoms of the three successive stages, viz. the inflammatory, the purulent, and lastly the third, when the pus extends and becomes diffused in different directions; sometimes causing description of the capsular ligament, and destruction of the bone; at other times leaving the joint intact. He alludes particularly to the frequently committed mistake of supposing the first stage to be an attack of common rheumatism, and earnestly urges upon his readers the importance of comparing the lengths of the two limbs, before they determine their diagnosis. In rheumatism no change is to be perceived, but whenever there is inflammation of the caxo-femoral joint, he assures us, from an extensive experience for the last thirty years, that there is invariably an elongation of the limb. However severe may be the pain, if only the swelling around the joint, and the elongation of the limb be absent, M. D. is satisfied that there can be no inflammation of the articular surfaces, and consequently no grounds for alarm. The seat of the inflammation at first is always exterior to the joint; it is neither in the bone itself, in the synovial membrane, fibro-cartilage, nor in the synovial glands, but invariably, according to the experience of M. D. is in the outer surface of the articular capsule, in the fibrous structures about it, and in the periosteum of the bone round the circumference of the acetabulum, and of the upper part of the femur itself. The limits within which the inflammatory process is usually at first circumscribed, are about one inch in extent all round the rim, or edge of the acetabular cavity; but the whole of this space is not generally affected simultaneously; the irritation may be concentrated either at one part of the capsule, or of the periosteum of the os ilii, or of the cervix femoris, or round the border of the acetabulum, or on the great trochanter.

By far the most common exciting cause is the local application of cold; and hence it is that in children it may frequently be traced to lying down on the damp grass when they are heated by play. A scrofulous diathesis indeed is often the root of the evil; but in very many cases there is not the slightest taint of that disease.

If accurately recognized and judiciously treated at first, we may almost always calculate upon a cure. Conjoined with entire rest, local bleedings, &c. M. D. urgently recommends the internal employment of emetic tartar in this, and indeed in all diseases which are induced by exposure to cold, and checked perspiration. He regards it as by far the most heroic of all antiphlogistic remedies. It is to be given in full doses and repeated every quarter of an hour, till the desired effects are obtained. He usually dissolves eleven grains in six ounces of water; and of this solution he orders one large tablespoonful at a time. Sometimes the addition of opium is necessary to induce the tolerance of the drug, or to counteract its effects upon the bowels.—*Algemeine Medic. Zeitung.*

---

PURULENT OPHTHALMIA OF INFANTS.

The treatment of this common and most dangerous affection adopted by M. Sanson at the Ophthalmological Clinic in the Hôtel Dieu, consists in the application of leeches and the use of other antiphlogistics until the purulent secretion commences; he then trusts chiefly to the external employment of astringents; and of these he prefers the nitrate of silver.

It is most effectual in the solid form; let the eye-lids be everted, and over their surfaces, after being wiped dry, let the caustic be freely applied. There is no occasion to wash the eye afterwards; the small quantity which may adhere will be dissolved in the tears, and will form an excellent collyrium. During the day, the following wash may be used frequently.

℞. Argenti nitrat. gr. iij.
Liquor plumbi, ♏vj.
Aquæ distill. ℥iv. M.

The system must be supported with mild, but nourishing food.

Mr. Wishart of Edinburgh praises highly the use of a solution of sulphate of zinc as a local application.

His formula is—

℞. Zinci sulphat. gr. xxiv.
Aquæ distill. ℥x. solve dim.
Adde Liquor plumbi, ʒss.
Tinct. camphoræ, ℥ss.—j.
Coletur, ut fiat collyrium.

Other British surgeons prefer an alum wash, containing from two to four grains of the salt to an ounce of water.

### ON THE RELATIONS OF THE CRANIUM TO THE ORGAN OF HEARING.

Professor Mojon, of Geneva, in a memoir lately read before the Royal Academy of Medicine, has suggested some novel and very interesting speculations on this subject. Hitherto we have been led to view the cranium only as a safe recipient of the cerebral mass and of its appendages; but M. M. ingeniously supposes that it serves at the same time as an harmonic case, or drum, to the auditory organs. Treviranus, Esser, and others had already observed that the tympanum is not essentially necessary to the transmission of sounds, and that the sonorous undulations may be conveyed to the nerve of hearing by the medium of the cranial bones; but no one, before our author, had attracted the attention of physiologists to the curious relations which seem to exist between certain states of the cranial bones and the power of discriminating musical sounds.

The post mortem examination of Dr. Bennati, first suggested to M. M. the following speculations, and they arose from his observing that the bones of the cranium were much thinner than usual, translucent at many points, and soldered together along the line of the sutures.

A similar condition of the cranial bones has subsequently been found by him in the body of another celebrated musician.

This coincidence of cranial attenuation, and musical endowments, led M. Mojon to consider whether it was possible that the one might be related to the other as cause and effect; and he has been induced by numerous observations to infer that the cranium is by no means quite passive, in the perception of sounds, that differences in the thickness of its walls may have very considerable influence in determining the degree of acuteness of the faculty, and therefore that it may be regarded as a sort of harmonic case which communicates its vibrations to the organs of hearing.

In confirmation of these views our author alludes to the cases of deaf people, who often can perceive very distinctly the sounds of a piano, or organ, by applying one extremity of an iron rod to their forehead, and the other to the instrument; and who may be made to hear what is said to them if only the voice is directed by a speaking trumpet upon some part of the forehead. It is not unfrequently also that a person whose hearing is indistinct, and who chances to wear a wig, can listen with much greater facility when the head is quite bare, than when it is covered.

The curious observations of M. Perier, on patients who had been trepanned, and who were found to hear quite distinctly any sound directed upon the cicatrix, even when both ears were effectually plugged, (vide our last No.) may also be mentioned as illustrative of M. Mojon's speculations.

Comparative anatomy shews that in a number of animals the transmission of sounds to the organ of hearing is assisted by numerous large sinuses, hollowed out in the bones of the cranium. To us it seems by no means improbable that the musical endowments of the feathered tribes may be in some degree at least modified, or influenced by the very attenuated condition of their cranial bones, and by the existence of the elastic lamellæ, which are found between their supernumerary cavities, as well as the passages or canals which extend into the labyrinth.

The only practical deduction from the preceding views, regards the assistance which may possibly be derived from attention to them, in our diagnosis of deafness, when we wish to discover whether it is owing to a palsied state of the auditory nerves themselves, or merely to some defect or injury of the adjunct members of the auditory apparatus. The every day occurrence of a person squeezing his head with both hands to deaden any very loud noise, may very probably effect the desired purpose, as well by interrupting the cranial vibrations, as by the direct obstruction of the auditory passages. —*Journ. Hebdomad.*

